# Off-target autophagy inhibition by SHP2 allosteric inhibitors contributes to their antitumor activity in RAS-driven cancers

**DOI:** 10.1172/JCI177142

**Published:** 2024-06-06

**Authors:** Yiming Miao, Yunpeng Bai, Jinmin Miao, Allison A. Murray, Jianping Lin, Jiajun Dong, Zihan Qu, Ruo-Yu Zhang, Quyen D. Nguyen, Shaomeng Wang, Jingmei Yu, Frederick Nguele Meke, Zhong-Yin Zhang

**Affiliations:** 1 Department of Medicinal Chemistry and Molecular Pharmacology and; 2 Department of Chemistry, Purdue University, West Lafayette, Indiana, USA.; 3 Departments of Internal Medicine, Pharmacology, and Medicinal Chemistry, University of Michigan, Ann Arbor, Michigan, USA.; 4 Institute for Cancer Research and; 5 Institute for Drug Discovery, Purdue University, West Lafayette, Indiana, USA.

**Keywords:** Oncology, Therapeutics, Cancer, Drug therapy, Phosphoprotein phosphatases

## Abstract

Aberrant activation of RAS/MAPK signaling is common in cancer, and efforts to inhibit pathway components have yielded drugs with promising clinical activities. Unfortunately, treatment-provoked adaptive resistance mechanisms inevitably develop, limiting their therapeutic potential. As a central node essential for receptor tyrosine kinase–mediated RAS activation, SHP2 has emerged as an attractive cancer target. Consequently, many SHP2 allosteric inhibitors are now in clinical testing. Here we discovered a previously unrecognized off-target effect associated with SHP2 allosteric inhibitors. We found that these inhibitors accumulate in the lysosome and block autophagic flux in an SHP2-independent manner. We showed that off-target autophagy inhibition by SHP2 allosteric inhibitors contributes to their antitumor activity. We also demonstrated that SHP2 allosteric inhibitors harboring this off-target activity not only suppress oncogenic RAS signaling but also overcome drug resistance such as MAPK rebound and protective autophagy in response to RAS/MAPK pathway blockage. Finally, we exemplified a therapeutic framework that harnesses both the on- and off-target activities of SHP2 allosteric inhibitors for improved treatment of mutant RAS–driven and drug-resistant malignancies such as pancreatic and colorectal cancers.

## Abstract 2

SHP2 allosteric inhibitors elicit off-target autophagy blockade that can be exploited for improved treatment of RAS-driven and drug-resistant cancers.

## Graphical abstract

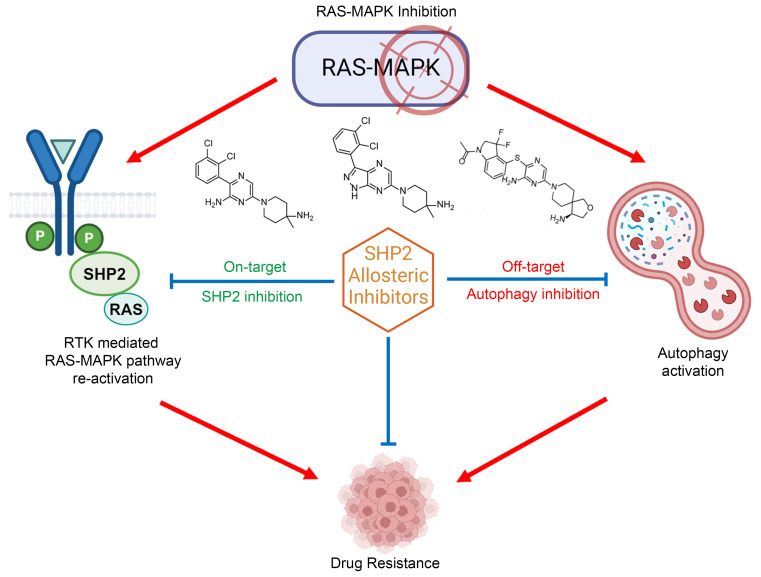

## Introduction

As membrane-bound small GTPases cycling between active GTP-bound and inactive GDP-bound states, the rat sarcoma (RAS) proteins serve as critical molecular switches in response to diverse extracellular stimuli, including growth factors (1). Activated RAS interacts with distinct effector proteins, thereby regulating diverse cytoplasmic signaling networks and cellular processes (1, 2). As one of the best-studied signaling pathways, the growth factor receptor–mediated RAS/RAF/MEK1/2/ERK1/2/MAPK cascade plays an essential role in cell proliferation, survival, and differentiation (3). Not surprisingly, this signaling process is aberrantly activated in 46% of all cancer types in The Cancer Genome Atlas data sets (4). Moreover, gain-of-function mutations in RAS rank among the most frequently observed genetic lesions in human cancers (1, 5), and the KRAS isoform alone is mutated in 9% of cancer cases, with high frequency in pancreatic ductal adenocarcinoma (72%), genomically stable colorectal cancer (69%), and non–small cell lung cancer (33%) (4). Accordingly, substantial efforts have been devoted to targeting the receptor tyrosine kinase (RTK)/RAS/MAPK pathway (6), yielding numerous small-molecule inhibitors of pathway components RTKs, KRAS, RAF, MEK1/2, and ERK1/2 that show efficacy in cancer patients (7–10). Unfortunately, the enthusiasm associated with this progress has been tempered by the emergence of various adaptive drug resistance mechanisms (11–17), which limits the effectiveness and/or duration of responses to these drugs.

Src homology 2 domain–containing phosphatase 2 (SHP2), encoded by *PTPN11*, plays a positive role downstream of RTKs to promote RAS activation (18, 19). SHP2 is autoinhibited by intramolecular interactions between its N-SH2 and phosphatase domains, which block substrate entrance to the active site. However, upon growth factor stimulation, the N-SH2 domain disengages with the phosphatase domain as a result of its preference for phosphorylated tyrosine motifs in RTKs and/or scaffold proteins, thus leading to an open and active conformation that is catalytically competent for SHP2 substrate turnover (20). Although the precise mechanism(s) underlying SHP2-mediated RAS activation is not fully understood (21), recent studies suggest that SHP2 acts upstream of the guanine nucleotide exchange factors SOS1 and SOS2 to stimulate RAS-GTP loading (22–27). Given this crucial role for SHP2 in RTK-induced RAS activation (18), there has been strong interest in developing SHP2 inhibitors as anticancer agents (21). Capitalizing on the unique aforementioned regulatory mechanism of SHP2, Novartis reported the first SHP2 allosteric inhibitor, SHP099, which binds to a tunnel formed at the interface of the N-SH2, C-SH2, and phosphatase domains and stabilizes SHP2 in its autoinhibited state (28). Subsequently, many SHP099-like SHP2 allosteric inhibitors (SHP2-AIs) have been disclosed (29) — including TNO155 (30), RMC-4550 (25), IACS-13909 (31), and JAB-3068 (WO 2017/211303 A1). Consistent with the role of SHP2 in the RTK/RAS/MAPK cascade, SHP2-AIs exhibit broad antitumor activity in RAS-dependent cancer models, such as those harboring RTK alterations and certain RAS and RAF mutations (23, 25, 28). Importantly, SHP2 inhibition also overcomes adaptive resistance to RAS/MAPK pathway inhibitors in multiple cancer models by interfering with RTK-mediated RAS reactivation (12, 22, 24, 25, 27, 30–35). Consequently, many SHP2-AIs in this class are being evaluated in phase I/II clinical trials for the treatment of solid tumors with elevated RTK activity and/or RAS mutations, either as a single agent or in combination with RAS or MEK1/2 inhibitors (36, 37).

In addition to the potential clinical applications of SHP2-AIs, they are also widely deployed as chemical probes to investigate the mechanisms by which SHP2 regulates cell physiology and contributes to human diseases, primarily on the premise that they inhibit SHP2 with exquisite specificity. Intriguingly, while SHP2-AIs are structurally related, share the same binding mode, and have comparable biochemical and cellular potencies, they exhibit a wide range of efficacies in cancers driven by aberrant RAS-MAPK activation (12, 22–25, 27, 28, 30–35). Moreover, some SHP2-AIs also display unexpected activity against the so-called RAS bypass cancers, which harbor RAS or RAF mutations known to be independent of upstream signaling from SHP2 (23, 26, 27, 33, 38). As part of our ongoing effort to interrogate SHP2 biology and to better define the antitumor activities elicited by SHP2-AIs, we compared several relatively well-studied SHP2-AIs in both cellular and mouse cancer models. To our surprise, we found that in addition to their on-target activity in blocking the RAS-induced ERK1/2 phosphorylation, these compounds also inhibit autophagy in a definitively SHP2-independent manner. This off-target effect casts a cautionary note on their use as chemical probes for SHP2 and suggests a therapeutic framework for improving treatment of RAS-MAPK mutant–driven cancers through intentional exploitation of off-target SHP2-AIs.

## Results

### Off-target autophagy blockade by SHP2-AIs.

**Figure 1:**
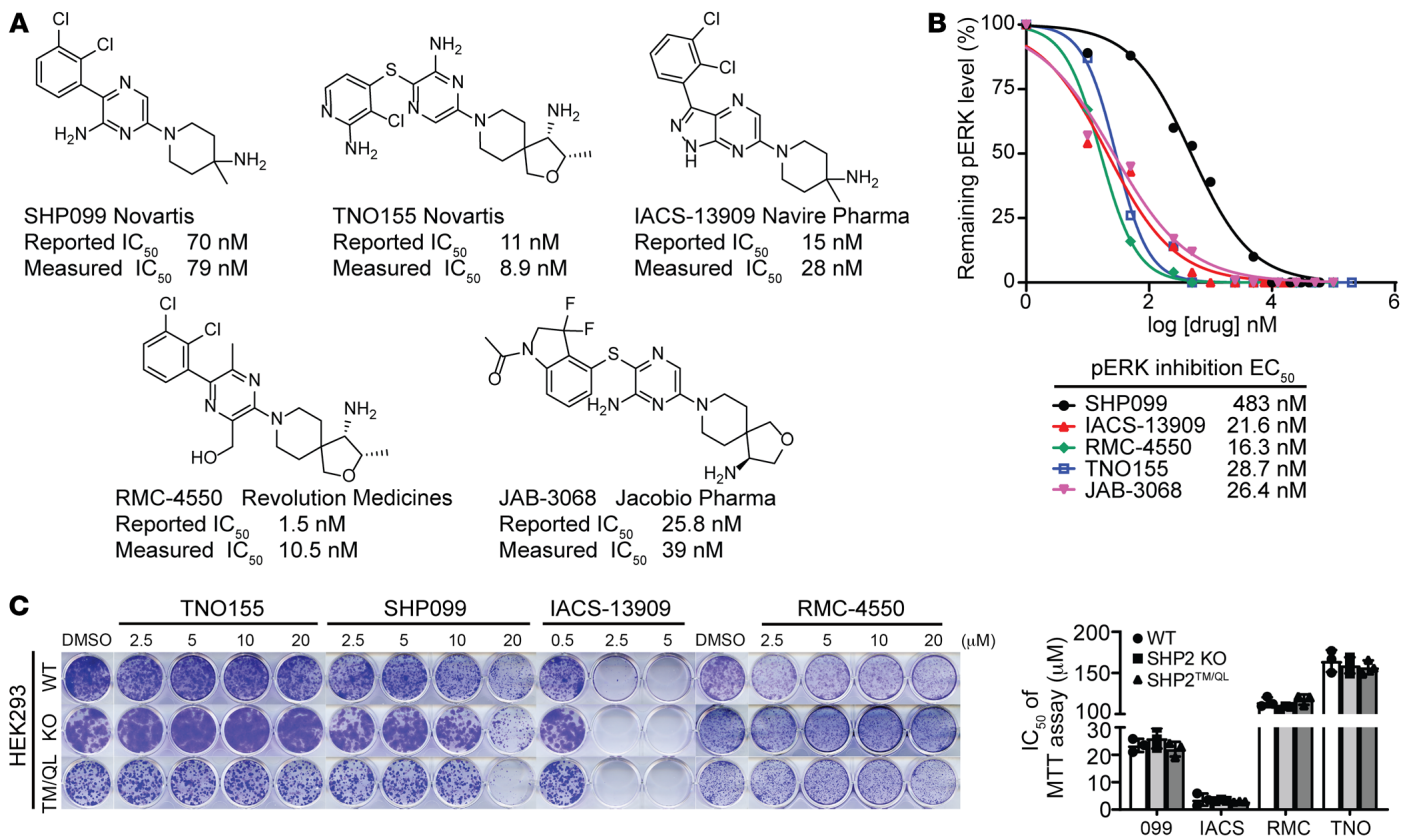
SHP2-AIs exert growth-inhibitory activity through an off-target mechanism. (**A**) Structures and reported or measured enzymatic inhibition activities of 5 representative SHP2-AIs. (**B**) SHP2 inhibition levels were determined by quantification of p-ERK level changes in Supplemental Figure 1A. To determine the EC_50_, the level of p-ERK after 10 μM SHP099 treatment was defined as 100% SHP2 inhibition. Representative data from 3 independent experiments displayed. (**C**) Result of colony formation assay and MTT assay using wild-type (WT), SHP2-KO, or SHP2^T253M/Q257L^ mutant–expressing HEK293 cells treated with indicated SHP2-AI for 10 days (colony formation) or 48 hours (MTT). Data are represented as means ± SD. TM, T253M; QL, Q257L.

SHP2-AIs such as SHP099 have increasingly been used as chemical probes to explore SHP2 biology and assess the therapeutic potential for targeting SHP2. Since the purpose of a chemical probe is to interrogate the intended target in complex living systems, it is essential that the probe molecule possess exquisite specificity and cellular activity against the target of interest. To more rigorously substantiate the specificity of SHP2-AIs for SHP2, we compared the effect of five SHP2-AIs — SHP099, TNO155, IACS-13909, RMC-4550, and JAB-3068 — in HEK293 cells (Figure 1A). The enzymatic IC_50_ values, both as reported and as measured in this study, were comparable for TNO155, IACS-13909, RMC-4550, and JAB-3068 (about 10–30 nM) and were several-fold lower than that of SHP099 (about 70–80 nM) (Figure 1A). The cellular efficacies, as measured by the EC_50_ values for the inhibition of ERK1/2 phosphorylation, were also similar for TNO155, IACS-13909, RMC-4550, and JAB-3068 (~20 nM) and were 24-fold lower than that of SHP099 (483 nM) (Figure 1B and Supplemental Figure 1A; supplemental material available online with this article; https://doi.org/10.1172/JCI177142DS1). Intriguingly, SHP099 and IACS-13909 displayed superior antiproliferative activities to those of TNO155 and RMC-4550 (Figure 1C), despite the fact that the latter two exhibited either more potent or comparable inhibitory activities toward SHP2 and ERK1/2 phosphorylation. Significant inhibition of colony formation was observed for SHP099 and IACS-13909 at 20 and 2.5 μM, respectively, while no effect was noted for TNO155 or RMC-4550 at concentrations up to 20 μM. SHP099 and IACS-13909 inhibited cell proliferation with an EC_50_ of about 23 and about 4 μM, respectively, while the EC_50_ values for TNO155- and RMC-4550–mediated cell growth inhibition were about 160 μM and about 110 μM. To ascertain whether the observed growth inhibition by SHP2-AIs was SHP2 dependent, we also evaluated these compounds in SHP2-CRISPR-deleted (SHP2-KO) HEK293 cells as well as in SHP2-KO HEK293 cells expressing SHP2^T253M/Q257L^, which is resistant to SHP2-AI binding (28). Surprisingly, these SHP2-AIs displayed the same antiproliferative activities in wild-type SHP2, SHP2-KO, and drug-resistant SHP2^T253M/Q257L^ cells (Figure 1C). These results indicate that the growth-inhibitory effects exerted by SHP2-AIs are not mediated by SHP2 inhibition, but rather through an unknown off-target mechanism.

**Figure 2:**
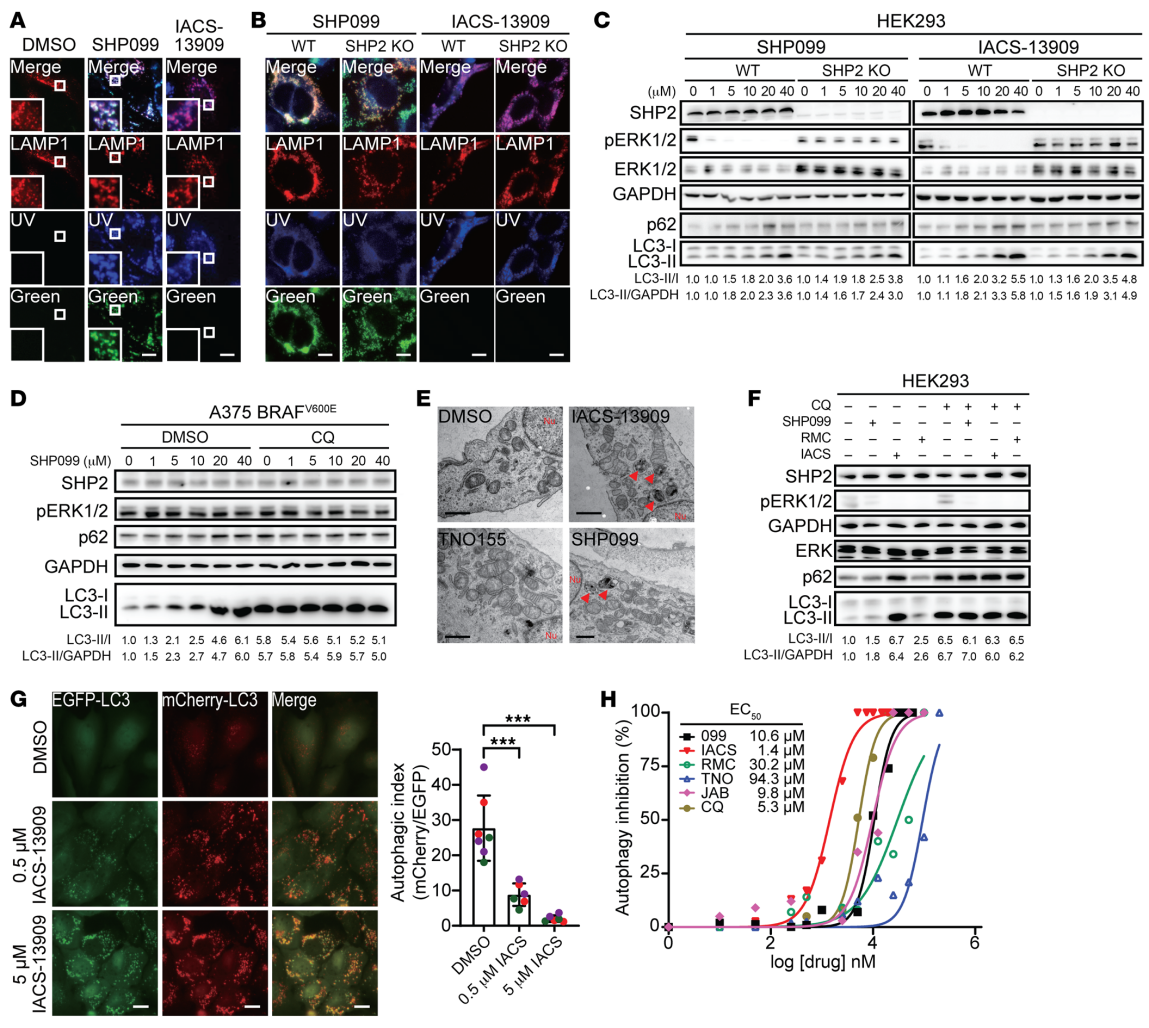
SHP2-AIs repress autophagy independent of SHP2. (**A**) RFP-LAMP1–expressing U2OS cells were treated with DMSO, 10 μM SHP099, or IACS-13909 for 3 hours. Representative images of LAMP1 (red, lysosome marker), SHP099 (green and blue), or IACS-13909 (blue) and merged channels are displayed. Scale bars: 10 μm. (**B**) RFP-LAMP1–expressing WT or SHP2-KO HEK293 cells were treated with 10 μM SHP099 or IACS-13909 for 3 hours. Representative images of LAMP1 (red, lysosome marker), SHP099 (green and blue), or IACS-13909 (blue) and merged channels are displayed. Scale bars: 10 μm. (**C**) WT or SHP2^–/–^ HEK293 cells were treated with a series of SHP099 or IACS-13909 concentrations for 6 hours. Total lysates were used for immunoblots. (**D**) A375 cells were treated with a series of SHP099 concentrations with or without the presence of 20 μM CQ for 6 hours. Total lysates were used for immunoblots. (**E**) U2OS cells were treated with 10 μM SHP099, TNO155, or IACS-13909 for 6 hours and visualized with transmission electronic microscopy. Scale bars: 1 μm. (**F**) HEK293 cells were treated with 10 μM SHP099, RMC-4550, or IACS-13909 for 6 hours with or without 20 μM CQ pretreatment for 3 hours. Total lysates were used for immunoblots. Lanes were run on the same gel but were noncontiguous. (**G**) EGFP-mCherry-LC3–expressing U2OS cells were treated with 0.5 or 5 μM IACS-13909 or DMSO for 6 hours. Representative images of EGFP (green, pH sensitive), mCherry (red, pH insensitive), and merged channels are displayed. Scale bars: 10 μm. Autophagic index indicates the ratio of the areas of mCherry^+^ puncta to EGFP^+^ puncta. Mean autophagic index is plotted, with each individual data point representing 1 analyzed cell field (5–10 fields total) from 3 independent experiments (labeled with different colors). Significance was determined by 1-way ANOVA followed by Dunnett’s multiple-comparison test. ***P < 0.001. (**H**) Autophagy inhibition levels were determined by quantification of LC3-II/I ratio changes in Supplemental Figure 1A. To determine the EC_50_, a 5-fold increase of LC3-II/I ratio in comparison with DMSO was defined as 100% autophagy inhibition. Representative data from 3 independent experiments displayed for all panels.

To further investigate this off-target mechanism, we noticed that cells treated with SHP099 or IACS-13909, but not TNO155 or RMC-4550, featured increased vacuolization. To determine whether the compound-induced vacuolization is related to the off-target effect, we incubated U2OS cells with 10 μM SHP099, IACS-13909, or TNO155. Unlike TNO155, treatment with SHP099 or IACS-13909 led to increased cytoplasm vacuolization, a typical morphological character of autophagy (39) (Supplemental Figure 1B). In fact, SHP099 and IACS-13909 accumulated on the vacuoles, as revealed by their autofluorescence properties (Supplemental Figure 1C). Since SHP2 is dispersed throughout the cell (40), the discrete vacuole localization of SHP099 or IACS-13909 suggests that they may have off-target binding partner(s) inside the cell. Indeed, SHP099 and IACS-13909 were found to colocalize with the lysosomal maker LAMP1 (41) (Figure 2A). Furthermore, by super-resolution structured illumination microscopy (42), we were able to observe IACS-13909 directly localized on the lysosomal membrane (Supplemental Figure 1D). Importantly, the compounds’ lysosomal localization is independent of SHP2 expression, since SHP099 and IACS-13909 also localized on the lysosomes in SHP2-deleted HEK293 cells (Figure 2B).

Autophagy is a biochemical process utilized by a cell to remove and recycle unnecessary or dysfunctional components in the cytoplasm through the formation of autophagosomes, which then fuse with lysosomes to execute the degradation of engulfed cargo by lysosomal enzymes (39). Given the importance of the lysosome in autophagy, we then evaluated the conversion of the autophagy marker LC3 from LC3-I to LC3-II to determine whether SHP099 or IACS-13909 disturbs the autophagy-lysosomal degradation pathway (43). Consistent with the vacuolization phenotype, SHP099 and IACS-13909 dose-dependently increased the LC3-II/I ratio and the level of the autophagic substrate p62, which correlates with the number of autophagosomes (43), in both wild-type and SHP2-KO HEK293 cells (Figure 2C). As a control, SHP2-AIs predictably attenuated ERK1/2 activity in wild-type but not SHP2-deleted HEK293 cells (Figure 2C). Together, these observations suggest that the SHP2-AIs SHP099 and IACS-13909 have the additional capability to modulate autophagy in an SHP2-independent manner. Moreover, SHP099 had no effect on phosphorylated ERK1/2 (p-ERK1/2) levels but dose-dependently increased the LC3-II/I ratio in constitutively active BRAF^V600E^ mutant A375 cells (Figure 2D). SHP099 did not further increase the LC3-II level in the presence of chloroquine, suggesting that the increased accumulation of LC3-II is due to inhibited autophagic flux. This is in accordance with the notion that BRAF^V600E^ drives ERK1/2 activation independent of upstream RAS regulation (44, 45) and indicates that SHP099-induced autophagosome accumulation is independent of MAPK pathway inhibition. To further corroborate these findings, transmission electronic microscopy was used to confirm the increased autophagosome formation upon treatment with 10 μM SHP099 and IACS-13909, but not 10 μM TNO155, in U2OS cells (Figure 2E).

Since the LC3-II level approximates the abundance of autophagosome, which can be induced by either autophagy activation or inhibition (43), we next determined the effects of SHP099, IACS-13909, and RMC-4550 on autophagic flux by analyzing LC3-II accumulation in the presence of the known autophagy inhibitor chloroquine (CQ) (46). These SHP2-AIs did not further increase the CQ-mediated LC3-II or p62 accumulation, indicating that they function to block autophagic flux (Figure 2F). To further substantiate this observation, we used a pH-sensitive mCherry-EGFP-LC3 reporter whose green, but not red, fluorescence is quenched when the reporter fuses with acidic lysosome (47). In a similar manner to both CQ and another autophagy inhibitor, the vacuolar H^+^-ATPase inhibitor bafilomycin A1 (Baf-A1) (48), treatment with SHP099 and IACS-13909 significantly increased the yellow puncta in U2OS cells, which is indicative of autophagy blockage (Figure 2G and Supplemental Figure 1E). Taken together, these data demonstrated that SHP099 and IACS-13909 act as late-stage autophagy inhibitors independent of SHP2.

To expand this finding to other SHP2-AIs, we also measured the ability of RMC-4550, TNO155, and JAB-3068 (Figure 1A) to promote LC3-II and p62 accumulation in HEK293 cells (Supplemental Figure 1A). Quantification of the LC3-II/I ratio as a function of compound concentration generated the EC_50_ values for autophagy inhibition by CQ (EC_50_ = 5.3 μM) and SHP2-AIs, ranging from 1.4 μM for IACS-13909 to 9.8 μM for JAB-3068, 10.6 μM for SHP099, 30.2 μM for RMC-4550, and 94.3 μM for TNO155 (Figure 2H). Similar dose-dependent autophagy inhibition by the SHP2-AIs was also observed in SHP2-KO HEK293 cells (Supplemental Figure 1F), again confirming that the off-target effect is SHP2 independent. The EC_50_ values for SHP2-AI–mediated autophagy blockage conform with their ability to induce autophagosome (Figure 2E) and yellow puncta formation (Figure 2G and Supplemental Figure 1E). Thus, among the SHP2-AIs, IACS-13909 exhibits the highest autophagy inhibition activity and is a nearly 4-fold more potent autophagy inhibitor than CQ. Notably, the EC_50_ values for autophagy inhibition by SHP099 (10.6 μM) and IACS-13909 (1.4 μM) were close to or even below the concentrations (≥10 μM for SHP099 and up to 3.3 μM for IACS-13909) routinely used to study their SHP2-dependent antitumor activities (12, 22–24, 27, 31–35, 38). These findings reveal that SHP2-AIs display SHP2-independent off-target inhibition of autophagy, and this off-target effect could confound their SHP2 on-target activities.

### Mechanism and structural basis of autophagy inhibition by SHP2-AIs.

**Figure 3:**
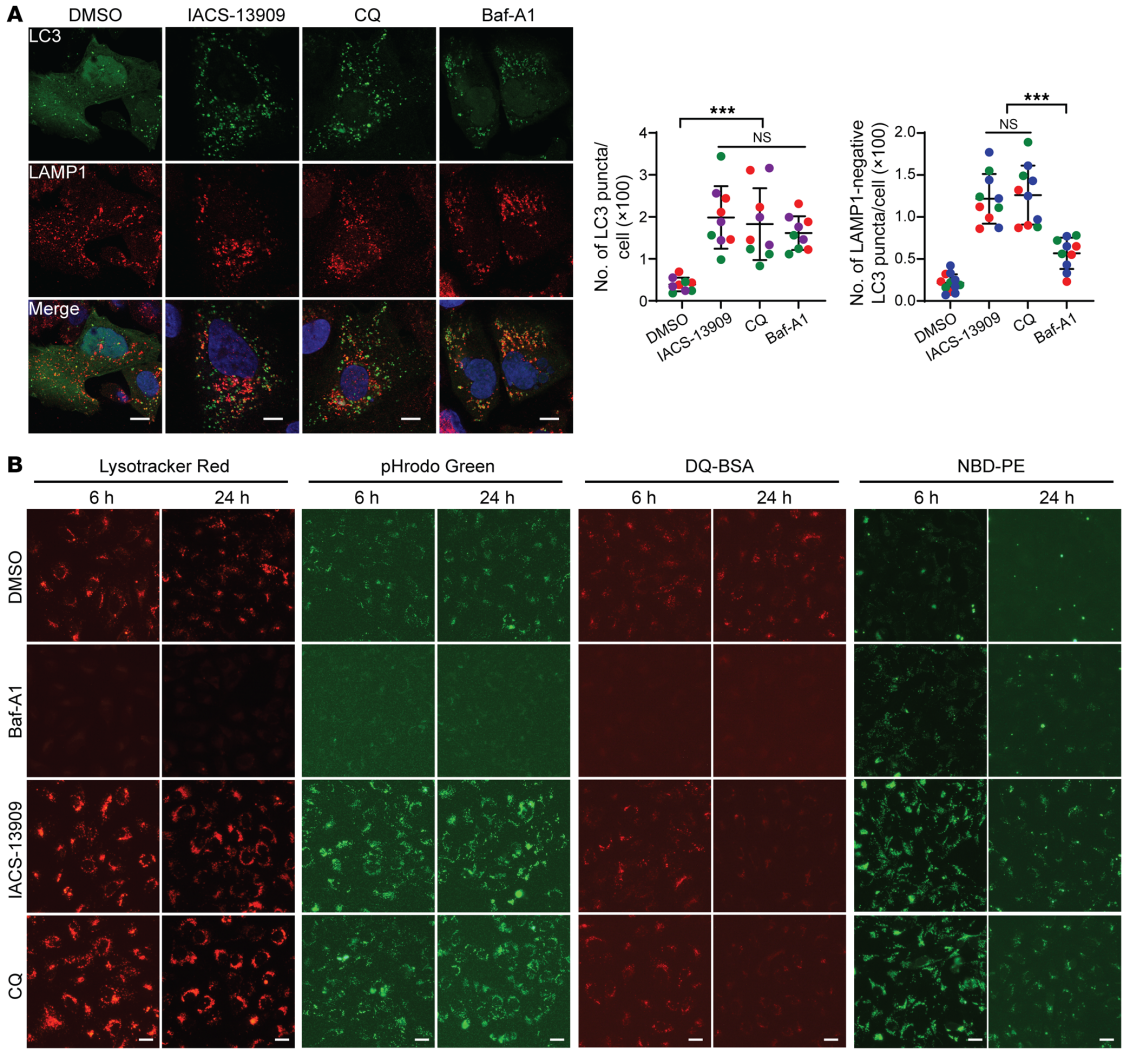
SHP2-AIs repress autophagy through impairing lysosome function. (**A**) GFP-LC3–expressing U2OS cells were treated with DMSO, 100 nM Baf-A1, 5 μM IACS-13909, or 10 μM CQ for 6 hours. Cells were fixed and stained with LAMP1 antibody for confocal imaging. LC3 accumulation was measured by counting of total LC3 (green) puncta and LC3 puncta without LAMP1 (red) colocalization. Scale bars: 10 μm. Data are represented as means ± SD from 3 different experiments (labeled with different colors). Significance was determined by 1-way ANOVA followed by Tukey’s multiple-comparison test. ***P < 0.001. (**B**) U2OS cells were labeled with LysoTracker Red, pHrodo Green, DQ-BSA, or NBD-PE and treated with DMSO, 100 nM Baf-A1, 5 μM IACS-13909, or 10 μM CQ for 6 or 24 hours. Scale bars: 10 μm. Representative data from 3 independent experiments displayed for all panels.

Late-stage autophagy inhibition can be effected by failed autophagosome-lysosome fusion and/or impaired lysosomal degradation (47). To define the mechanism behind SHP2-AI–mediated autophagy inhibition, we first examined whether these compounds could perturb autophagosome-lysosome fusion. We compared IACS-13909 with 2 late-stage autophagy inhibitors: Baf-A1 (48) and CQ (46). Baf-A1 obstructs proton transport and neutralizes the otherwise acidic lysosomal pH, which impedes lysosomal degradation, whereas CQ can impair autophagosome-lysosome fusion (46). Confocal imaging of U2OS cells treated with DMSO, Baf-A1, IACS-13909, or CQ revealed that Baf-A1, IACS-13909, and CQ all promoted accumulation of LC3 puncta, in line with their ability to inhibit autophagy (Figure 3A). In agreement with a recent report (46), CQ, but not Baf-A1, led to reduced colocalization of LC3 with LAMP1 and decreased autophagosome-lysosome fusion. Like CQ, IACS-13909 also diminished colocalization of LC3 with LAMP1, indicative of its ability to block autophagosome-lysosome fusion (Figure 3A).

In addition to interfering with the autophagosome-lysosome fusion process, CQ may also limit autophagic flux through inhibition of lysosomal cargo degradation (49, 50). To examine whether SHP2-AIs can also disrupt lysosomal functions, we used LysoTracker Red and pHrodo Green Dextran as lysosomal pH indicators, as they exhibit increased fluorescent intensity in acidic environments (51). As shown in Figure 3B, the decreased lysosomal acidification mediated by Baf-A1 significantly reduced LysoTracker Red and pHrodo Green signals. On the contrary, CQ does not have a significant effect on lysosomal pH upon prolonged administration (52). In fact, an increase in LysoTracker fluorescence was observed upon CQ treatment (Figure 3B), which is consistent with the finding that CQ-mediated lysosomal stress activates lysosome biogenesis (53). Like CQ, IACS-13909 did not affect lysosomal pH and instead demonstrated increased fluorescence (Figure 3B), likely due to a similar induction of lysosomal biogenesis, which was confirmed through increases in LAMP1 and CTSB staining (54) (Supplemental Figure 2A). We next compared the effects of Baf-A1, CQ, and IACS-13909 on lysosomal degradation. DQ-BSA Red, a self-quenching protein substrate that produces bright red fluorescence upon hydrolysis, was used as a readout for protein degradation, whereas nitrobenzoxadiazole-conjugated phosphoethanolamine (NBD-PE), a fluorescent phospholipid surrogate, was used to quantify phospholipid degradation (55, 56). As expected, Baf-A1 completely blocked DQ-BSA hydrolysis but only partially inhibited NBD-PE degradation (Figure 3B), which is in line with previous reports that lysosomal proteases are more sensitive to changes in pH than phospholipases (57, 58). In contrast, CQ and IACS-13909 strongly inhibited phospholipase activity but had little effect on protease activity (Figure 3B). Taken together, these results demonstrated that IACS-13909 inhibits autophagy through blockage of autophagosome-lysosome fusion and impairment of lysosomal phospholipid degradation.

**Figure 4:**
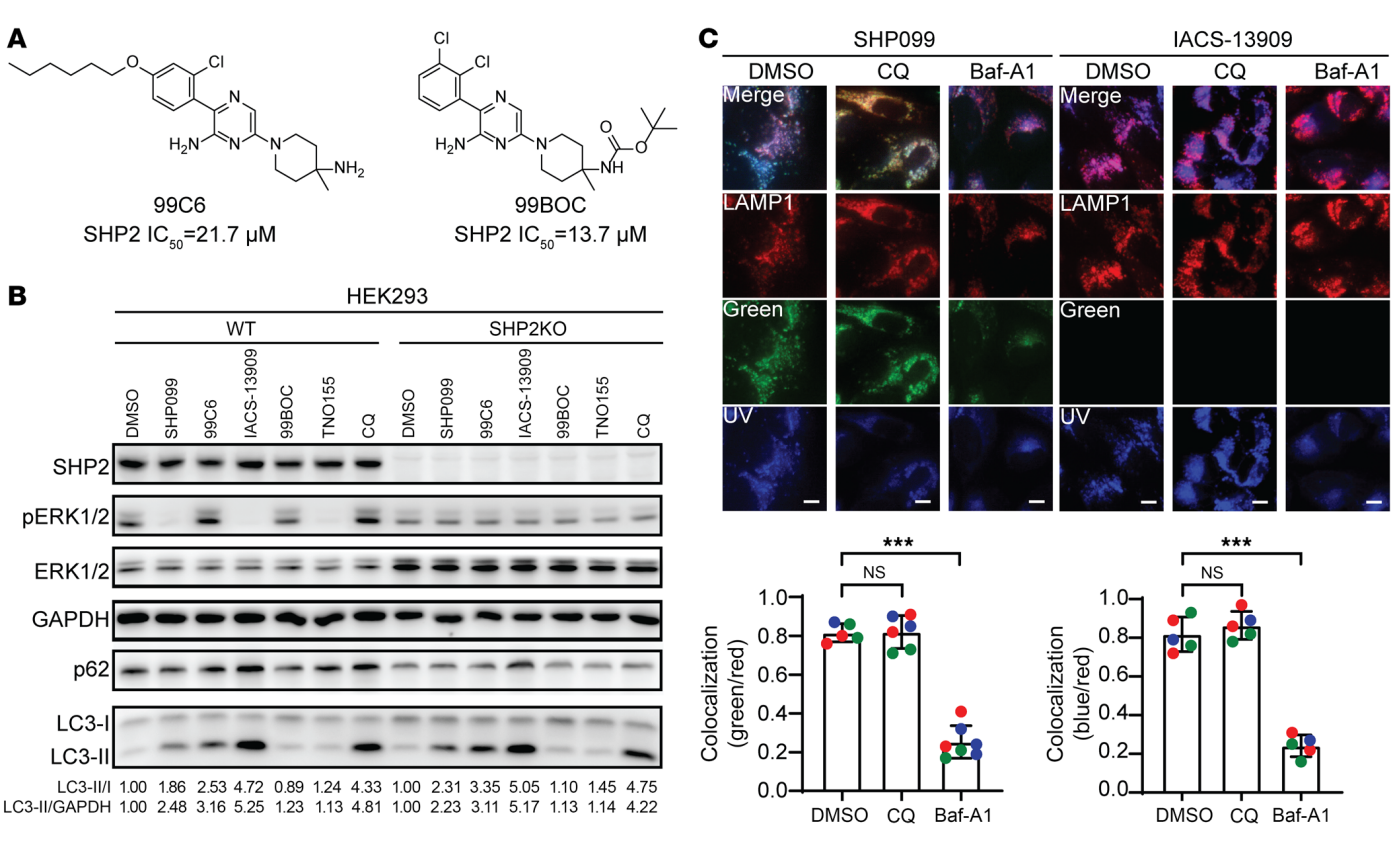
The structural basis of off-target autophagy inhibition by SHP2-AIs. (**A**) Structure of SHP099 derivatives 99C6 and 99BOC. (**B**) WT or SHP2-KO HEK293 cells were treated with 10 μM of indicated compounds for 6 hours. Total lysates were used for immunoblots. (**C**) RFP-LAMP1–expressing U2OS cells were treated with DMSO, 100 nM Baf-A1, or 10 μM CQ for 6 hours and then treated with 10 μM SHP099 for 3 hours. Representative images of LAMP1 (red, lysosome marker), SHP099 (green and blue), and merged channels are displayed. Scale bars: 10 μm. Colocalization index indicates the ratio of the areas of green/blue^+^ to red (LAMP1)^+^ puncta. Mean colocalization index is plotted, with each individual data point representing 1 analyzed cell field (5–10 fields total) from 3 independent experiments (labeled with different colors). Data are represented as means ± SD. Significance was determined by 1-way ANOVA followed by Dunnett’s multiple-comparison test. Representative data from 3 independent experiments displayed for all panels. ***P < 0.001.

Since SHP2-AIs and CQ appear to inhibit autophagy through similar mechanisms, we surmised that they may share key structural features important for autophagy inhibition. To that end, we recognized that SHP2-AIs (and CQ) structurally resemble cationic amphiphilic drugs, which contain a hydrophilic amine head group that can be protonated in acidic compartments, and a hydrophobic tail consisting of an aromatic or aliphatic ring that can anchor in lipid bilayers of cellular membranes (50). Molecules of this class are known to accumulate in lysosomes and inhibit phospholipid degradation (49, 50, 55, 59). To understand the structural basis of SHP2-AI–mediated off-target autophagy inhibition, we prepared 2 derivatives of SHP099, 99C6 and 99BOC (Figure 4A), and evaluated their ability to inhibit both SHP2 and autophagy. Since the 2,3-dichlorophenyl group of SHP099 is known to make extensive hydrophobic interactions with the side chains of Leu254, Gln257, Pro491, and Gln495 in SHP2 (28), it followed that removal of the 3-chloro group and addition of a hexyloxy group at the 4 position of the phenyl ring in SHP099 dramatically diminished 99C6’s ability to inhibit SHP2 phosphatase activity and thus ERK1/2 phosphorylation inside the cell (Figure 4, A and B, and Supplemental Figure 2B). Interestingly, 99C6 exhibited even more potent autophagy inhibition activity, presumably due to its increased hydrophobicity (Figure 4B and Supplemental Figure 2B), further demonstrating that the autophagy inhibition off-target effect is SHP2 independent. The crystal structure of SHP2 in complex with SHP099 also revealed a key hydrogen bond interaction between the backbone carbonyl of SHP2 Phe113 and the basic amine group of SHP099 (28). Not surprisingly, *tert*-butyloxycarbonyl (BOC) protection of the amine in SHP099 (99BOC) abolished its capacity to inhibit both SHP2 activity and autophagy, owing to the loss of the essential hydrogen bond for SHP2 binding and elimination of the basicity required for lysosomal retention, respectively (Figure 4, A and B, and Supplemental Figure 2C). Finally, we showed that the lysosomal targeting activity of SHP099 and IACS-13909 depends on the acidic pH in the lysosome (pH = 4.5), since pretreatment of the cell with Baf-A1 obliterated SHP099 and IACS-13909 localization on the lysosomes, while treatment by CQ, which does not raise lysosomal pH, failed to affect their colocalization with LAMP1 (Figure 4C). Taken together, these results unraveled the structural basis of off-target autophagy inhibition by SHP2-AIs. They also demonstrated that SHP2-AIs block autophagic flux by decreasing autophagosome-lysosome fusion and impeding lysosomal phospholipid degradation.

### Off-target autophagy inhibition contributes to SHP2-AI’s anticancer activity.

**Figure 5:**
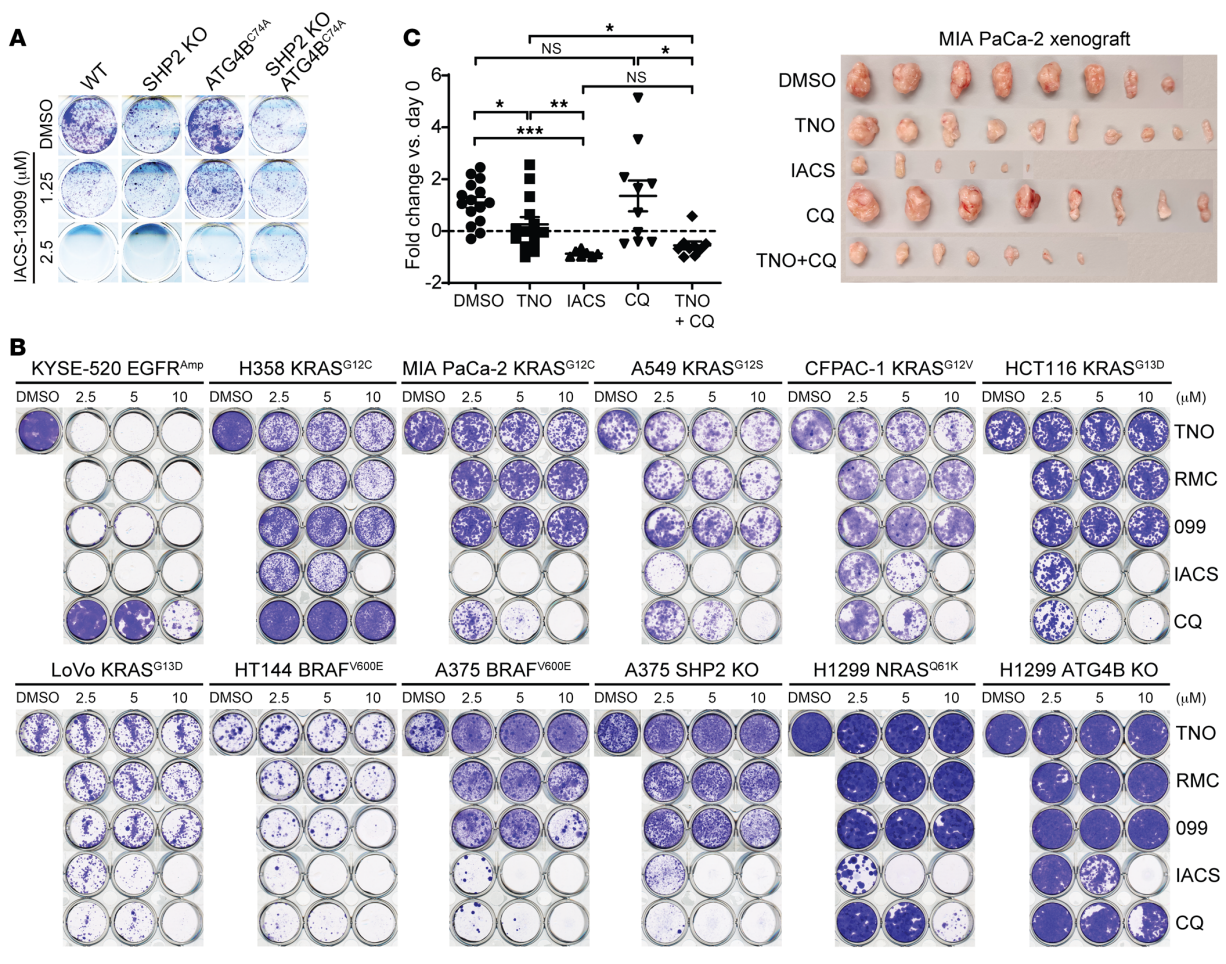
Autophagy inhibition contributes to the antitumor activity of SHP2-AIs. (**A**) Results of colony formation assay using WT or SHP2^–/–^ HEK293 cells with or without overexpression of the dominant-negative ATG4B^C74A^ mutation and treated with DMSO or 1.25 or 2.5 μM IACS-13909 for 10 days. (**B**) Results of colony formation assay using a panel of cancer cell lines treated with DMSO or indicated compounds for 10 days. (**C**) Changes in tumor volume of MIA PaCa-2 xenografts on day 21 compared with day 1 after treatment with vehicle control, 50 mg/kg TNO155, 50 mg/kg IACS-13909, 50 mg/kg CQ, and CQ with TNO155 (*n* = 6–10 per treatment group). Data are represented as means ± SD. Significance was determined by Brown-Forsythe and Welch’s ANOVA test followed by 2-stage linear step-up procedure of Benjamini, Krieger, and Yekutieli. *P < 0.05; **P < 0.01; ***P < 0.001. Representative data from 3 independent experiments displayed for **A** and **B**.

Given the importance of autophagy for optimal tumor growth and survival (60–62), we speculated that off-target autophagy inhibition may contribute to the anticancer activity of SHP2-AIs. To further demonstrate that the cell growth–inhibitory effects exerted by IACS-13909 and SHP099 also rely on autophagy impairment, we obliterated autophagic capabilities in both wild-type and SHP2-KO HEK293 cells via expression of a dominant-negative ATG4B^C74A^ mutant (63) (Supplemental Figure 3A). Only cells that were devoid of both SHP2 and autophagy were able to survive IACS-13909 treatment at 2.5 μM (Figure 5A), which is within the concentration range used in previous studies (31). These findings indicate that IACS-13909’s autophagy inhibition effects clearly contribute to its cell growth–inhibitory activity.

To further substantiate that SHP2-independent autophagy inhibition contributes to SHP2-AI’s antitumor activity, we analyzed a series of cell lines bearing *EGFR^AMP^*, *KRAS^G12^*, *KRAS^G13^*, *NRAS^Q61^*, or *BRAF^V600^* genetic mutations, which are known to display differential dependence on upstream RTK/SHP2 signaling. All known KRAS^G12^ mutants, including KRAS^G12C^ and KRAS^G12S^, possess decreased intrinsic GTPase activity, GAP-mediated GTP hydrolysis, and intrinsic GDP/GTP exchange, and therefore exhibit an increased reliance on SOS1/2 for nucleotide cycling. On the other hand, NRAS^Q61H/K^ and KRAS^G13D^ mutants exhibit severely compromised GTP hydrolysis and wildly enhanced nucleotide exchange, respectively, and hence do not depend on SOS1/2 for RAS activation (25, 44, 64). Similarly, the BRAF^V600E^ mutant functions as constitutively active monomers that decouple BRAF from RAS-GTP activity (44, 45). As a result, KRAS^G12^ mutant cells were predicted to be partially sensitive to SHP2 inhibition, whereas KRAS^G13^, NRAS^Q61^, and BRAF^V600^ mutant cells were not expected to be responsive to SHP2 inhibition. To determine the SHP2 dependency of these cell lines, we evaluated TNO155 at concentrations that display significant inhibition of SHP2, but not of autophagy (Figure 1B and Figure 2H), and measured dose-dependent p-ERK1/2 levels after 4 and 48 hours (Supplemental Figure 3B). Consistent with the notion that SHP2 acts upstream of SOS1/2 to regulate RAS-GTP loading (22–27), phosphorylation of ERK1/2 in EGFR-amplified KYSE-520 cells was highly sensitive to SHP2 inhibition, while ERK1/2 phosphorylation in KRAS^G12^ mutated cells showed only partial sensitivity to SHP2 inhibition. Importantly, the KRAS^G12C^ inhibitor AMG510 (65) — but not TNO155, RMC-4550, nor the validated SHP2 degrader D26 (66) — completely abrogated ERK1/2 activation in KRAS^G12C^ MIA PaCa-2 pancreatic cancer cells, indicating that the incomplete p-ERK1/2 inhibition by TNO155 and RMC-4550 in KRAS^G12C^ mutant cells was likely due not to inadequate drug efficacy but rather to a limited reliance on SHP2 (Supplemental Figure 3C). As predicted, cells expressing KRAS^G13D^ (HCT 116 and LoVo), NRAS^Q61K^ (H1299), or BRAF^V600E^ (A375 and HT144) were insensitive to SHP2 inhibition (Figure 5B and Supplemental Figure 3B). Together, these observations are in full agreement with the known dependencies of RAS and RAF mutants on upstream RTK/SHP2 signaling.

After establishing the SHP2 dependencies of our cell line series, we performed colony formation assay with TNO155, RMC-4550, SHP099, IACS-13909, and CQ to determine whether the antitumor properties of SHP2-AIs are SHP2 and/or autophagy dependent. All SHP2-AIs were quite effective in inhibiting KYSE-520 cell growth, but in KRAS^G12^ mutated cells, TNO155, RMC-4550, or SHP099 treatment led to only partial growth inhibition, while IACS-13909 and CQ led to a complete response (Figure 5B). Remarkably, SHP099 has a nearly 10-fold lower potency for SHP2 compared with TNO155 and RMC-4550 (Figure 1A), so the observed partial growth inhibition by SHP099 likely arose from a combination of SHP2 and autophagy inhibition. As predicted, in cancer cells carrying BRAF^V600E^, KRAS^G13D^, or NRAS^Q61K^ mutations, treatment with up to 10 μM TNO155 or RMC-4550 showed little or no growth inhibition, thus validating the SHP2 independence of these cell lines (Figure 5B). Intriguingly, in the SHP2-independent BRAF^V600E^ cell lines (A375 and HT144), SHP099 and IACS-13909 generated significant inhibition of cell growth only at concentrations with substantial autophagy inhibition activity (Figure 2H), which is consistent with the observation that BRAF^V600E^-driven cancer cells are particularly sensitive to autophagy inhibition (67). To authenticate this important observation, we deleted SHP2 in BRAF^V600E^ A375 cells and found similar growth-inhibitory effects after SHP099 and IACS-13909 treatment (Figure 5B), which further confirmed that these observed antitumor effects are SHP2 independent and likely due to autophagy inhibition. Indeed, the autophagy inhibitor CQ suppressed both parental and SHP2-KO BRAF^V600E^ A375 cell growth with similar efficacies (Figure 5B). Strikingly, IACS-13909, which displays comparable in vitro SHP2 inhibitory activity to TNO155 and RMC-4550, completely suppressed colony formation across the board, irrespective of a cell line’s SHP2 dependency status (Figure 5B), indicating that its potent autophagy inhibition activity contributes to its cell growth inhibition. The autophagy dependency of IACS-13909–mediated cell growth inhibition is further supported by the observed positive correlation between the IC_50_ of IACS-13909– and CQ-mediated inhibition of colony formation in both SHP2-independent and partially dependent cancer cells (Supplemental Figure 3D). Moreover, growth inhibition by IACS-13909 and CQ was strongly attenuated when the essential autophagy gene *ATG4B* was deleted in NRAS^Q61K^ H1299 cells (Figure 5B), further verifying that autophagy inhibition is responsible for the strong antitumor effects of IACS-13909. Finally, we analyzed the observed sensitivity of cancer cell lines to either SHP2 deletion or SHP2 inhibition (23) and found that the two SHP2-independent cell lines with strong sensitivity to SHP099 treatment, pancreatic cancer cell line T3M4_PANCREAS (KRAS^Q61H^) and melanoma cell line WM115_SKIN (BRAF^V600E^), are highly dependent on autophagy (*ATG4B*) for survival (DepMap Portal; https://depmap.org/portal) (Supplemental Figure 3E). Collectively, these results support the conclusion that off-target autophagy inhibition by SHP2-AIs contributes to their antitumor activity.

Pancreatic cancer, a malady notorious for its resistance to treatment, harbors a high prevalence of KRAS mutations and thus calls for the development of RAS/MAPK pathway inhibitors (4, 11). While tumors derived from abnormal regulation of the RAS/MAPK pathway have been shown to display increased autophagic flux (68, 69), it was recently demonstrated that treatment of pancreatic cancer cells with RAS-MAPK inhibitors elicits further elevation of autophagy (70, 71). This finding emphasizes the role of autophagy in adaptive resistance mediated by RAS-MAPK inhibition, but also exposes a vulnerability of cancer cells to concurrent inhibition of autophagy. Consequently, we postulated that SHP2-AIs with pronounced off-target autophagy inhibition (e.g., SHP099 and IACS-13909) may be more effective than more selective SHP2 inhibitors (e.g., TNO155 and RMC-4550) in the treatment of cancers addicted to autophagy. We tested this hypothesis using MIA PaCa-2 (KRAS^G12C^) xenografts. Once tumors reached 100 mm^3^, mice were treated daily with vehicle control, TNO155, IACS-13909, CQ, or a combination of CQ and TNO155. No significant side effects were observed, as evidenced by the limited weight loss and lack of appreciable change in animal histopathology (Supplemental Figure 4, A and C). In support of our hypothesis, TNO155 alone caused more than 70% reduction of tumor size, but IACS-13909 treatment led to nearly 80% tumor regression in all mice (Figure 5C and Supplemental Figure 4C). In addition, although autophagy inhibition by CQ alone had no significant effect on tumor growth, combined treatment with TNO155 and CQ produced a synergistic effect (~50% tumor regression) that was comparable to that of the IACS-13909 single-agent treatment (Figure 5C and Supplemental Figure 4C). Immunohistochemistry analyses confirmed that while both TNO155 and IACS-13909 could reduce ERK1/2 phosphorylation, only IACS-13909 treatment led to autophagy inhibition, as evidenced by the accumulation of autophagy adaptor protein p62 (72), and a subsequent rise in the apoptotic indicator cleaved caspase-3, which is a functional consequence of impaired autophagy (73) (Supplemental Figure 4B). Although CQ alone yielded comparable apoptosis and p-ERK1/2 levels to vehicle control, the combination of TNO155 and CQ led to increased apoptosis and reduced p-ERK1/2 levels comparable to those of IACS-13909 treatment alone (Supplemental Figure 4B). These findings are consistent with previous reports that combination of MAPK pathway and autophagy inhibition results in higher apoptosis and antitumor efficacy (70, 71). Taken together, our data demonstrate that SHP2-AI–mediated off-target autophagy inhibition contributes to the antitumor activity of SHP2-AIs both in vitro and in vivo and suggest a well-rounded approach to targeting adaptive resistance driven by RAS-MAPK inhibition.

### Harnessing SHP2-AIs’ off-target effect for improved treatment of RAS-mutated cancers.

While RAS/MAPK pathway inhibitors have made major strides in cancer treatment, drug-induced reactivation of MAPK signaling is a key constraint in clinical efficacy (7, 12–17). Given the role of SHP2 in RTK-induced RAS activation, SHP2-AIs have been utilized in combination with RAS/MAPK pathway inhibitors to overcome this adaptive resistance (22, 27, 33–35, 74). Unfortunately, further reinforcement of MAPK pathway inhibition also activates autophagy to protect cancer cells from death (70, 71, 75). In light of these observations, there is increasing interest in targeting autophagy in combinatorial cancer treatment. Indeed, although hydroxychloroquine (HCQ), an analog of CQ with equipotency for autophagy inhibition (76), had limited efficacy for pancreatic cancer patients (77), the combination of CQ or HCQ with inhibitors of the MAPK pathway (e.g., the MEK1/2 inhibitor trametinib) has generated promising results in preclinical models of both KRAS-mutant pancreatic cancer and NRAS-mutant melanoma (70, 71). As a result, a phase I clinical trial (NCT03825289, ClinicalTrials.gov) has been initiated to investigate the efficacy of trametinib plus HCQ for the treatment of patients with metastatic or locally advanced pancreatic ductal adenocarcinoma.

**Figure 6:**
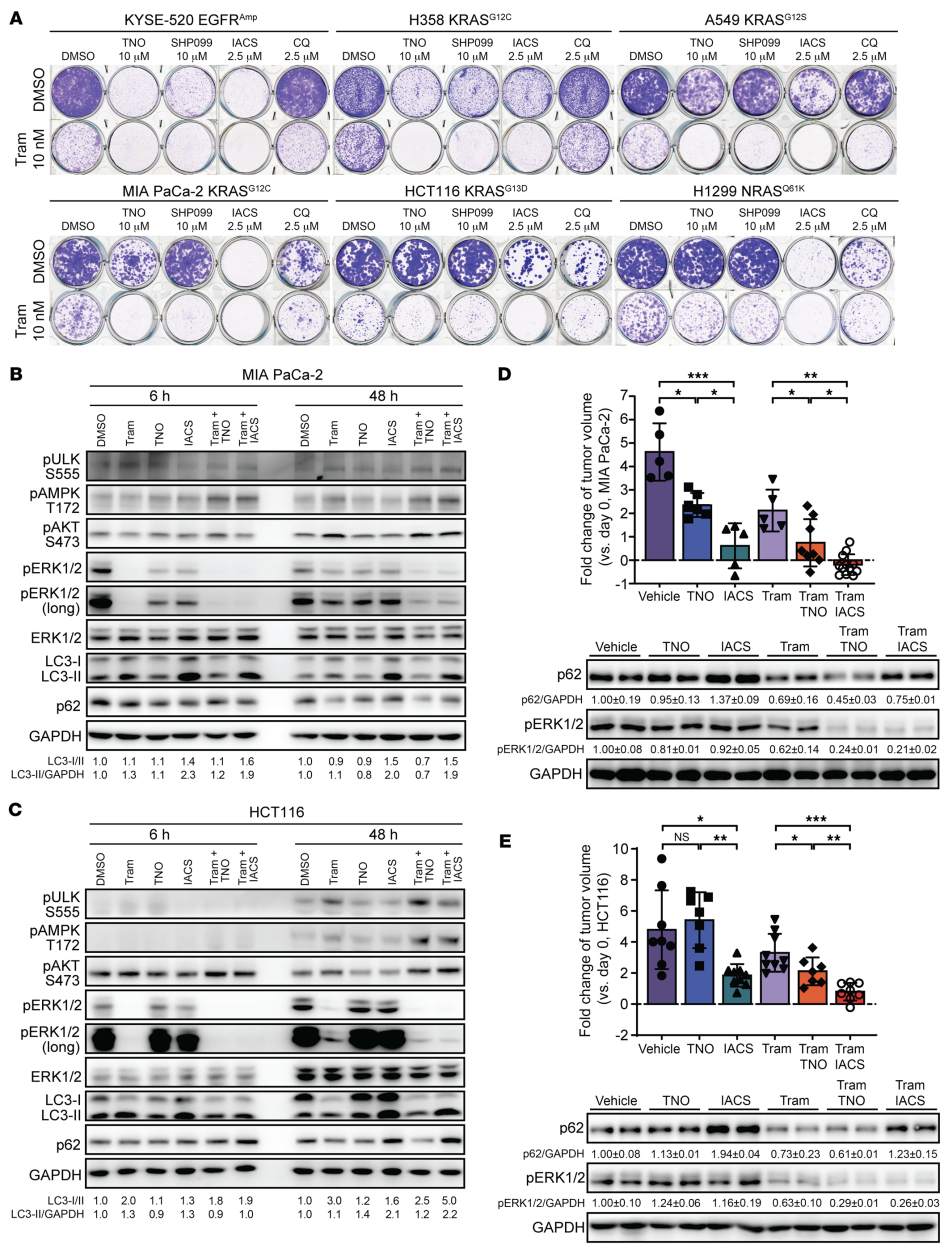
MEK/SHP2/autophagy triple inhibition is highly effective in KRAS-mutated cancers. (**A**) Results of colony formation assay using a panel of cancer cell lines treated with DMSO or indicated compounds for 10 days. (**B** and **C**) MIA PaCa-2 (**B**) or HCT 116 (**C**) cells were treated with DMSO, 10 nM trametinib, 1 μM TNO155, or IACS-13909 or combinations for the times indicated. Total lysates were used for immunoblots. (**D** and **E**) Changes in tumor volume of MIA PaCa-2 (**D**) and HCT 116 (**E**) xenografts on day 21 compared with day 1 after treatment with vehicle control, 40 mg/kg TNO155, 40 mg/kg IACS-13909, 0.25 mg/kg trametinib, or combinations (*n* = 5–10). Total lysates from individual tumors were used for immunoblots. Data are represented as means ± SD. Significance was determined by Brown-Forsythe and Welch’s ANOVA test followed by 2-stage linear step-up procedure of Benjamini, Krieger, and Yekutieli. *P < 0.05; **P < 0.01; ***P < 0.001. Representative data from 3 independent experiments displayed for **A**–**C**.

Based on our findings and discussions above, we predicted that the combined use of SHP2 and RAS/MAPK pathway inhibitors would also elicit this protective autophagy, and further, employment of an SHP2-AI with off-target autophagy inhibition properties would generate considerable benefits for cancer treatment. To that end, we first conducted colony formation assay with a panel of cancer cell lines to evaluate the antitumor effects induced by the combination of SHP2-AIs with the MEK1/2 inhibitor trametinib. Consistent with previous reports (24, 26, 27, 33–35, 70, 71), combination of trametinib with either the SHP2 inhibitor TNO155 or the autophagy inhibitor CQ led to synergistic growth inhibition in a panel of cancer cell lines that display varied sensitivity to SHP2 inhibition (Figure 6A and Supplemental Figure 5A). Interestingly, combination of trametinib with IACS-13909, even at a 4-fold lower concentration of TNO155, produced comparable or more powerful growth inhibition than trametinib combined with either TNO155, SHP099, or CQ, indicating that potent autophagy inhibition by IACS-13909 may synergize with its SHP2-inhibitory effects (Figure 5, A and C) and overall make a strong contribution toward improving the efficacy of RAS/MAPK pathway inhibition (Figure 6A and Supplemental Figure 5A).

To define the effects of SHP2-AIs on the protective autophagy induced by the MEK1/2 inhibitor trametinib, we treated MIA PaCa-2 (KRAS^G12C^) and HCT 116 (KRAS^G13D^) cells with SHP-AIs or trametinib alone or in combination. In agreement with earlier studies (24, 26, 27, 33–35), SHP2 inhibition in MIA PaCa-2 cells partially inhibited ERK1/2, but not AKT, phosphorylation and blocked prolonged MEK1/2 inhibition–mediated p-ERK1/2 rebound after 48 hours (Figure 6B and Supplemental Figure 5B). Although ERK1/2 phosphorylation in HCT 116 cells appeared to be unchanged by SHP2 inhibition, we found that SHP2-AI treatment could still block the p-ERK1/2 rebound induced by MEK1/2 inhibition, an observation that is consistent with the role of SHP2 as a common mediator of RTK-induced RAS/MAPK pathway reactivation (Figure 6C and Supplemental Figure 5B). As shown previously (70, 71), prolonged inhibition of MEK1/2 by trametinib increased autophagy in both cell lines, which was accompanied by enhanced ULK and AMPK phosphorylation (Figure 6, B and C). Combination of trametinib with the SHP2-AI TNO155 or IACS-13909 led to a further increase in p-ULK^S555^ and p-AMPK^T172^ levels in comparison with those triggered by trametinib treatment alone, thus signifying an even stronger induction of protective autophagy in the presence of SHP2-AIs (Figure 6, B and C). As expected, IACS-13909 treatment alone or in combination with trametinib resulted in increased blockage of autophagic flux and accumulation of LC3-II and p62 (Figure 6, B and C). These data confirmed that p-ERK1/2 rebound and autophagy induction, the previously reported adaptive resistance mechanisms to MEK1/2 and/or SHP2 inhibition, are driving MIA PaCa-2 and HCT 116 cancer cell growth and suggest that the combination of IACS-13909 with trametinib can obliterate both SHP2-dependent and -independent drug resistance mechanisms.

To harness both the on-target and off-target effects of SHP2-AIs for improved therapeutic targeting of abnormal RAS/MAPK pathway activation, we hypothesized that triple inhibition of MEK1/2, SHP2, and autophagy through treatment with trametinib and IACS-13909 in combination would be highly efficacious in KRAS-driven tumors, as both the drug treatment–induced p-ERK1/2 rebound and autophagy induction would be eliminated simultaneously. To determine whether the trametinib and IACS-13909 combination indeed has superior antitumor activity, we treated MIA PaCa-2 and HCT 116 xenograft mice with vehicle, 40 mg/kg IACS-13909 or TNO155, 0.25 mg/kg trametinib, or combinations of 0.25 mg/kg trametinib with 40 mg/kg IACS-13909 or TNO155 when the tumor size reached 250 mm^3^. The various combination treatments did not cause significant body weight change (Supplemental Figure 6A). For MIA PaCa-2 xenografts, when compared with vehicle control, trametinib alone was able to reduce tumor growth by more than 50%, while the combination of trametinib with TNO155 led to a visible improvement and almost 85% reduction in tumor growth (Figure 6D and Supplemental Figure 6B). In support of our prediction, IACS-13909 was more efficacious than TNO155 in repressing tumor growth (~86% reduction for IACS-13909 vs. ~49% reduction for TNO155), but more substantially, the combination of IACS-13909 with trametinib led to an approximately 11% regression in tumor size as compared with the approximately 85% reduction in tumor growth for the trametinib and TNO155 combination (Figure 6D and Supplemental Figure 6B), thus demonstrating a superior combination for the treatment of MIA PaCa-2 pancreatic cancer cells. Consistent with our in vitro results (Figure 6A), HCT 116 tumors were largely insensitive to TNO155. However, TNO155 was able to improve the efficacy of trametinib (~30% tumor reduction for trametinib alone vs. ~53% reduction in combination with TNO155), likely through the abolishing of the p-ERK1/2 rebound mechanism (Figure 6E and Supplemental Figure 6B). Strikingly, IACS-13909 alone was highly effective in reducing the tumor size by approximately 61%, likely owing to a sufficient combination of both SHP2 and off-target autophagy inhibition (Figure 6E and Supplemental Figure 6B). Moreover, the administration of trametinib in combination with IACS-13909 resulted in an approximately 82% reduction in tumor size (Figure 6E and Supplemental Figure 6B), thus confirming the effectiveness of MEK1/2/SHP2/autophagy triple inhibition in resensitizing RAS-MAPK–driven cancer cells that become otherwise insensitive to RAS-MAPK inhibition.

The above results demonstrate that IACS-13909 is superior to TNO155 in combination with MEK1/2 inhibition because of the addition of autophagy inhibition plus SHP2 inhibition. To further determine whether there is any benefit of adding SHP2 inhibition to MEK1/2 plus autophagy inhibition, we also compared trametinib plus CQ versus the treatment group that received the more selective SHP2 inhibitor TNO155 plus trametinib and CQ. As shown in Supplemental Figure 6, D–F, SHP2 or autophagy inhibition alone using TNO155 or CQ significantly improved the efficacy of trametinib in attenuating MIA PaCa-2 xenograft growth. Importantly, the trametinib/CQ/TNO155 triple combination was more efficacious than the trametinib/CQ double combination in suppressing tumor growth in vivo. These results indicate that there is an added benefit of SHP2 inhibition over MEK1/2 plus autophagy inhibition. Western blot and immunohistochemical analyses of tumors from the MIA PaCa-2 and HCT 116 xenografts confirmed that TNO155 and IACS-13909 yielded similar on-target SHP2 inhibition, as shown by the comparable decrease in p-ERK1/2 and Ki67 (Figure 6, D and E, and Supplemental Figure 5C). Consistent with its superior antitumor activity, IACS-13909 indeed evoked additional off-target autophagy inhibition and subsequent induction of apoptosis, as manifested by the upregulation of p62 and cleaved caspase-3, respectively (Figure 6, D and E, and Supplemental Figure 5C). Collectively, our results show that simultaneous targeting of MEK1/2, SHP2, and autophagy can not only inhibit oncogenic RAS/MAPK signaling but also overcome 2 major drug-induced resistance mechanisms: the RTK-mediated feedback reactivation of RAS and the upregulation of autophagy resistance mechanisms. Our results also tangibly demonstrate that the combination of trametinib with IACS-13909 can be highly effective in the treatment of RAS-MAPK–driven cancers.

## Discussion

As a central node downstream of various RTKs and upstream of RAS, SHP2 is a highly sought-after target for cancer drug discovery (21). Numerous SHP2-AIs have been described, and more than ten SHP2-AIs are now in clinical development for the treatment of cancers caused by aberrant RAS-MAPK activation, either as single agents or in combination with inhibitors of RTKs, KRAS, or MEK1/2 (36, 37). These SHP2-AIs have been touted as exceptionally specific for SHP2, even though some of them, specifically SHP099 and IACS-13909, have exhibited unexpected antitumor activities in tumors driven by autonomous RAS or RAF mutations that bypass the SHP2 requirement (23, 26, 27, 33, 38). While one early study noted that the effects of SHP099 on MAPK signaling could not be explained solely by on-target SHP2 inhibition (78), no systematic study has since been conducted to examine the existence and identity of SHP2-AI–mediated off-target effects.

We discovered that SHP2-AIs possess a previously unrecognized off-target activity. By using SHP2-AI–resistant, SHP2-independent, and SHP2-KO cell lines, we firmly established that SHP2-AIs inhibit autophagy in an SHP2-independent manner. Among the SHP2-AIs examined, IACS-13909 and SHP099 are endowed with the most pronounced autophagy inhibition activity with EC_50_ values of 1.4 and 10.6 μM, respectively. In fact, IACS-13909 inhibits autophagy with a nearly 4-fold greater potency than CQ, a widely used autophagy inhibitor. Mechanistically, SHP2-AIs anchor directly onto the lysosomal membrane and disrupt lysosome function by hindering phospholipid metabolism and instigating lysosomal disorganization. Structure and activity studies reveal that SHP2-AIs behave similarly to cationic amphiphilic drugs, such as CQ, that are known to accumulate in intracellular compartments, such as lysosomes, where they inhibit lipid processing and provoke phospholipidosis in cells, tissues, and organs (49, 50, 55, 59). Indeed, it has been reported that one indication of SHP099 toxicity is phospholipidosis in the liver (30).

Because SHP099 has been well regarded as an SHP2-specific molecule, it has also been extensively used as a chemical probe to interrogate SHP2 biology. However, SHP099 and IACS-13909 exhibit significant off-target autophagy inhibition at concentrations commonly used to study SHP2-dependent functions (12, 22–24, 27, 31–35, 38), effectively suggesting that conclusions from earlier works should be revisited and future studies using these inhibitors as SHP2-specific tools should be conducted with caution. Although SHP2-AIs fall short of expectations as SHP2-specific chemical probes, we ascertained whether their off-target effects could be harnessed through polypharmacology to achieve optimal therapeutic benefits. To that end, tumor cells are known to upregulate and rely on autophagy to support their metabolism, growth, and survival (60–62). Thus, the therapeutic targeting of autophagy has been explored as a potential strategy for cancer treatment, specifically in the context of pancreatic cancer. Although the addition of CQ to MEK1/2 or ERK1/2 inhibition increased the therapeutic response in multiple KRAS-driven tumor models (70, 71), the observed potency of these combinations is still modest. In addition, CQ and its analog hydroxychloroquine are associated with undesirable side effects and extensive toxicity profiles, including cardiotoxicity, ocular toxicity, and neuromyotoxicity (60, 79). Prior studies of SHP099 were carried out at plasma concentrations greater than 10 μM (75–100 mg/kg oral dosing daily), which are well beyond the threshold required to significantly inhibit autophagic flux; thus the observed antitumor effects were not due exclusively to SHP2 inhibition (22, 23, 27, 28). We demonstrated that the off-target autophagy inhibition activity of SHP2-AIs largely contributes to their antitumor activity. By comparing the therapeutic efficacies of IACS-13909 and TNO155, we also showed that as single agents, SHP2-AIs possessing off-target autophagy inhibition exhibit superior anticancer activity to those with more selective SHP2 inhibition properties.

To explore the translatability of our findings to improve the therapeutic efficacy of targeted RAS/MAPK pathway blockade, we focused on RAS mutant–driven cancers, which are often addicted to autophagy for survival (61). Recent works reveal that targeted interventions to various RAS/MAPK pathway components inevitably lead to rapid development of 2 types of adaptive resistance: the RTK-mediated RAS/MAPK pathway rebound (7, 12–17, 61) and autophagy activation (70, 71, 75), both of which limit drug efficacy. In agreement with previous observations (12, 22, 24, 25, 27, 30–35), we firmly established that SHP2 inhibition can block the RTK-mediated rebound in MAPK signaling. We also confirmed that not only does MEK1/2 inhibition upregulate autophagy but, also, coinhibition of MEK1/2 and SHP2 further elevates autophagy, together indicating that autophagy induction as a resistance mechanism cannot be avoided when multiple components of the RAS/MAPK pathway are targeted. Consequently, there is a growing consensus that combination therapy, rather than RAS/MAPK pathway inhibitor monotherapy, is essential in achieving favorable clinical benefits. To that end, we showed that SHP2-AIs harboring both on-target SHP2 inhibition and off-target autophagy inhibition can simultaneously overcome both types of adaptive drug resistance. We determined that molecules such as IACS-13909 provide an improved therapeutic framework in the treatment of cancers with a dependence on dysregulated RAS/MAPK signaling through cotargeting of both oncogenic signaling and its accompanying adaptive resistance mechanisms: the MAPK pathway rebound and autophagy activation. Indeed, IACS-13909, either alone or in combination with trametinib, displays substantially more potent antitumor activity than TNO155 in RAS- and RAF-mutant cell lines as well as MIA PaCa-2 (KRAS^G12C^) and HCT 116 (KRAS^G13D^) xenograft tumor models. Although there are multiple clinical trials investigating combinations of MEK1/2 and SHP2 inhibition or MEK1/2 and autophagy inhibition, no study has yet evaluated the efficacy of MEK1/2/SHP2/autophagy triple inhibition. Our study illustrated that such a triple inhibition strategy is highly effective in the suppression of tumor growth and superior to both MEK1/2/SHP2 and MEK1/2/autophagy cotherapies. In summary, the unexpected and fortuitous polypharmacology of IACS-13909 may provide a more effective therapeutic opportunity for targeting the oncogenic RAS/MAPK pathway and improving clinical outcomes in patients with RAS mutant–driven malignancies, including those with pancreatic, colorectal, and lung cancer.

## Methods

### Sex as a biological variable.

Both male and female mice (approximately 1:1) were used in this study. However, sex was not considered as a biological variable.

### Cell lines and reagents.

All cell lines were obtained from ATCC unless specified otherwise. HEK293 (CRL-1573), H1299 (CRL-5803), HCT 116 (CCL-247), U2OS (HTB-96), A375 (CRL-1619), MIA PaCa-2 (CRM-CRL-1420), CFPAC-1 (CRL-1918), HT144 (HTB-63), and MDA-MB-231 (CRM-HTB-26) were grown in DMEM (Corning Cellgro, 10-013-CV), while H358 (CRL-5807), KYSE-520 (Leibniz Institute DSMZ, ACC 371), and LoVo (CCL-229) cells were grown in RPMI 1640 (Corning Cellgro, 10-040-CV) supplemented with 10% FBS (Gibco, 26400044), penicillin (50 U/mL), and streptomycin (50 μg/mL) (Corning, MT30002CI) in a 37°C incubator containing 5% CO_2_. Cells were seeded at 40%–80% confluence in antibiotic-free medium and grown overnight. Transfection was performed using polyethylenimine (Polysciences, 23966-2), Lipofectamine 2000 (Invitrogen, 11668019), or RNAiMAX (Invitrogen, 13778075) according to the manufacturer’s recommendations.

SHP099 (CT-SHP099), IACS-13909 (CT-IACS-13909), RMC-4550 (CT-RMC4550), JAB-3068 (CT-JAB3068), and TNO155 (CT-TNO155) were purchased from Chemietek.

### Clonogenic survival assays.

Cells (100 to 500) were seeded in 6- or 12-well plates 1 day before treatment with DMSO or indicated drugs, allowed to grow until they formed colonies (7–14 days), rinsed twice with PBS to remove floating cells, fixed in 4% formaldehyde in PBS for 15 minutes, and stained in 0.1% crystal violet/10% ethanol for 20 minutes. Staining solution was aspirated, and colonies were washed with water 3 times, air-dried, and visualized with scanner (EPSON PERFECTION V700 PHOTO). At least 3 biological replicates were performed.

### Immunoblotting.

Whole-cell lysates were generated in modified radioimmunoprecipitation (RIPA) buffer (50 mM Tris-HCl [pH 8.0], 150 mM NaCl, 2 mM EDTA, 1% NP-40, and 0.1% SDS) and supplemented with protease inhibitors (40 μg/mL PMSF, 2 μg/mL antipain, 2 μg/mL pepstatin A, 20 μg/mL leupeptin, and 20 μg/mL aprotinin) and phosphatase inhibitors (10 mM NaF, 1 mM Na_3_VO_4_, 10 mM β-glycerophosphate, and 10 mM sodium pyrophosphate). Total protein lysate was resolved by standard SDS-PAGE and transferred to nitrocellulose membrane in 1× transfer buffer and 20% methanol. Membranes were incubated with their respective primary and HRP-conjugated secondary antibodies and visualized using an Azure imaging system. Antibodies against p–p42/44 MAPK (p-Erk1/2) (Thr202/Tyr204) (4370; 1:2,000), GAPDH (97166; 1:5,000), p42/44 MAPK (Erk1/2) (4695; 1:3,000), p-ULK1 (Ser555) (5869; 1:1,000), p-AMPKα (Thr172) (2535; 1:1,000), and p-AKT (Ser473) (4060; 1:3,000) were obtained from Cell Signaling. LC3 (L7543; 1:3,000) and p62 (P0067; 1:3,000) antibodies were obtained from Millipore. SHP2 (sc-7384; 1:500) antibody was purchased from Santa Cruz Biotechnology. Quantifications were performed using ImageJ (NIH).

### Imaging and immunofluorescence.

Cells expressing pDEST-CMV mCherry-GFP-LC3B WT (Addgene, 123230; deposited by Robin Ketteler), LAMP1-RFP (Addgene, 1817; deposited by Walther Mothes), and EGFP-LC3 (Addgene, 11546; deposited by Karla Kirkegaard) were used to examine the cellular localization of corresponding genes. Cells were cultured directly on glass coverslips in 12- or 24-well plates. After experiments, cells were fixed with 4% paraformaldehyde in PBS (Corning, 20-031-CV) for 15 minutes at room temperature, permeabilized with 0.2% Triton X-100 in PBS for 10 minutes, and blocked with BSA (Sigma-Aldrich, A2153). For immunofluorescence, CTSB antibody (Cell Signaling, 31718; 1:400) was applied overnight at 4°C, followed by washing with TBST and 1 hour of incubation with appropriate secondary antibody. DNA staining (0.5 μg of Hoechst no. 33258 per mL; Sigma-Aldrich, 94403) was used to identify cell nuclei. After washing with PBS, the coverslips were mounted with Vectashield Antifade mounting Medium (Vector Laboratories, H-1000-10). Images were obtained with a Nikon Inverted Microscope Eclipse Ti-S (Nikon Instruments) or Zeiss LSM 880 confocal microscope.

To examine drug localization, LAMP1-RFP–expressing U2OS cells were treated with 10 μM SHP099 (green and blue fluorescence) or IACS-13909 (blue fluorescence) for 3 hours. Live cell images were taken by a Nikon Inverted Microscope Eclipse Ti-S (Nikon Instruments). For super-resolution imaging, LAMP1-RFP–expressing U2OS cells were cultured directly on glass coverslips in 24-well plates and treated with IACS-13909 (blue fluorescence) for 3 hours. Cells were fixed with 4% paraformaldehyde in PBS and mounted with antifade mounting solution for Nikon Super Resolution N-SIM system imaging.

For transmission electronic microscopy imaging, U2OS cells were fixed in 2.5% glutaraldehyde in 0.1 M cacodylate buffer overnight at 4°C and then washed 3 times for 5 minutes each with 0.1 M sodium cacodylate buffer. After washing, samples were further fixed with 1% OsO_4_ (Sigma-Aldrich, 201030) and 0.8% FeCN (Sigma-Aldrich, P3289) for 1 hour, then washed 3 times for 5 minutes each with water. Samples were then stained with 2% uranyl acetate in water for 20 minutes and subsequently rinsed with water 3 times for 5 minutes each. Dehydration was performed through grades of ethanol (50%–100%). After dehydration, samples were infiltrated with acetonitrile and embedded in resin. Ultrathin sections (60 nm) were cut using a Leica UC7 ultramicrotome (Leica Microsystems Inc.) and visualized under a Tecnai T12 transmission electron microscope (Thermo Fisher Scientific) at an accelerating voltage of 80 kV.

To examine lysosomal functions, U2OS cells were treated with DMSO, 100 nM bafilomycin A1, 5 μM IACS-13909, or 10 μM CQ for 6 hours or 24 hours. LysoTracker Red DND-99 (Thermo Fisher Scientific, L7528; 50 nM) was applied to the cells 30 minutes before imaging. pHrodo Green Dextran (Thermo Fisher Scientific, P35368; 100 μg/mL), DQ Red BSA (Thermo Fisher Scientific, D12051; 20 μg/mL), or NBD-PE (Thermo Fisher Scientific, N360; 7.5 μM) was applied to the cells along with the drug treatment (6 or 24 hours before imaging). Images were obtained with a Nikon Inverted Microscope Eclipse Ti-S (Nikon Instruments).

### Xenograft experiments.

All animal experiments were approved by the Purdue University Institutional Animal Care and Use Committee. MIA PaCa-2 and HCT 116 xenografts were established by subcutaneous injection of 1 × 10^6^ cells in 100 μL 50% Matrigel (Corning) into the right and left flanks of male and female (1:1) nude mice (NU/J, The Jackson Laboratory, 002019) when animals were 8–10 weeks of age.

When tumors reached 100–250 mm^3^ as measured by calipers (volume = ½ length × width^2^), mice were randomized into groups (5–10 mice per group).

For the MIA PaCa-2 model (Figure 3C), mice were treated through oral gavage with (a) vehicle, (b) TNO155, (c) IACS-13909, (d) CQ, or (e) CQ/TNO155 combination. The following oral gavage dosing regimens were used: TNO155 50 mg/kg bid, IACS-13909 50 mg/kg qd, or CQ 50 mg/kg qd.

For MIA PaCa-2 and HCT 116 models (Figure 4, D and E), mice were treated through oral gavage with (a) vehicle, (b) TNO155, (c) IACS-13909, (d) trametinib, (e) trametinib/TNO155 combination, or (f) trametinib/IACS-13909 combination. The following oral gavage dosing regimens were used: TNO155 40 mg/kg bid, IACS-13909 40 mg/kg qd, trametinib 0.25 mg/kg qd.

All drugs were resuspended in 0.6% methylcellulose, 0.5% Tween-80, and 0.9% saline. Caliper and weight measurements were performed every other day and continued until termination of the experiments.

### Histology.

Tissues were fixed in 4% paraformaldehyde (Thermo Fisher Scientific, 28908), incubated overnight at 4°C, embedded in paraffin, serially sectioned (7 μm), and stained with H&E according to standard methods. For immunohistochemistry, deparaffinized and hydrated sections were subjected to antigen retrieval by boiling in 10 mM sodium citrate for 20 minutes. Sections were then incubated with p62 (Millipore, P0067; 1:400), p–p42/44 MAPK (p-Erk1/2) (Thr202/Tyr204) (Cell Signaling, 4370; 1:150), Ki67 (Cell Signaling, 12202; 1:300), and cleaved caspase-3 (Cell Signaling, 9661; 1:400) antibodies at 4°C overnight. Signals were developed using DAB substrate (Vector Laboratories, SK-4100) and detected by VECTASTAIN Elite ABC kit. Images were captured with a Nikon Inverted Microscope Eclipse Ti-S (Nikon Instruments).

The synthesis of SHP2-AI derivatives 99C6 and 99BOC and the cloning, expression, and purification of SHP2 protein and SHP2 allosteric inhibition assay are described in Supplemental Methods.

### Statistics.

Unless specified otherwise, graphs in figures display individual values and mean ± SD. *P* values less than 0.05 were considered statistically significant. Statistical analyses were performed in GraphPad Prism. Significance was determined by 1-way ANOVA followed by Dunnett’s multiple-comparison test or Tukey’s multiple-comparison test, or Brown-Forsythe and Welch’s ANOVA test followed by 2-stage linear step-up procedure of Benjamini, Krieger, and Yekutieli for multiple comparison, as indicated in the figure legends.

### Study approval.

All animal experiments were approved by the Purdue University Institutional Animal Care and Use Committee.

### Data availability.

Data supporting the findings of this study are available within the figures and supplemental material of the article. The Supporting Data Values file includes all raw data points used for the figures. All raw data associated with figures and extended data are available upon request.

## Author contributions

YB conceptualized the study. YM and YB conceived the experiments and interpreted the data. YM, YB, and AAM performed the cellular experiments. YM and YB performed the in vivo experiments. JM, JL, and QDN designed and synthesized the compounds and derivatives. JD and ZQ performed enzymatic assays. RYZ generated critical cell lines. SW provided key reagents. JY and FNM contributed to the revision. YM and YB wrote the initial draft of the manuscript. ZYZ supervised the study, contributed to the design and interpretation of all experiments, and reviewed and wrote the manuscript with input from all coauthors. The order of authors, including co–first authors, was determined based on contributions to the overall design, experimentation, analyses, and writing.

## Supplementary Material

Supplemental data

Unedited blot and gel images

Supporting data values

## Version 1 06/06/2024

In-Press Preview

## Version 2 08/01/2024

Electronic publication
