# Supplementary material for: Off-target autophagy inhibition by SHP2 allosteric inhibitors contributes to their antitumor activity in RAS-driven cancers
Source: J Clin Invest. 2024 Jun 6;134(15):e177142. doi: 10.1172/JCI177142 (PMC11291269; doi:10.1172/JCI177142)
Supplement: Supplemental data [file jci-134-177142-s245.pdf]

Title: Off-target autophagy inhibition by SHP2 allosteric inhibitors contributes to their anti-tumor activity in RAS-driven cancers

Authors: Yiming Miao[1#], Yunpeng Bai[1#*], Jinmin Miao[1], Allison A. Murray[1], Jianping Lin[1], Jiajun Dong[1], Zihan Qu[2], Ruo-Yu Zhang[1], Quyen Nguyen[2], Shaomeng Wang[5], Jingmei Yu[1], Frederick Nguele Meke[1], and Zhong-Yin Zhang[1,2,3,4*]

Affiliations:
[1]Department of Medicinal Chemistry and Molecular Pharmacology, Purdue University; West Lafayette, IN 47907, USA.
[2]Department of Chemistry, Purdue University; West Lafayette, IN 47907, USA.
[3]Institute for Cancer Research, Purdue University; West Lafayette, IN 47907, USA.
[4]Institute for Drug Discovery, Purdue University; West Lafayette, IN 47907, USA.
[5]Departments of Internal Medicine, Pharmacology, and Medicinal Chemistry, University of Michigan; Ann Arbor, MI 48109, USA.

[#]These authors contributed equally to the work.

*Correspondence: Yunpeng Bai, 201S University Street, West Lafayette, IN 47907, (765)-496-7449, bai62@purdue.edu and Zhong-Yin Zhang, 720 Clinic Drive, West Lafayette, IN 47907, (765)-496-3720, zhang-zy@purdue.edu

The supplemental file includes:
Supplemental Materials and Methods

Supplemental Figures 1-6

Supplemental Materials and Methods

Synthesis of SHP2-AI derivatives 99C6 and 99BOC

All reagents were purchased from commercial suppliers and used without further purification. Thin-layer chromatography was performed using Merck glass plates precoated with silica gel 60 F254. Column chromatography was performed using KP-SIL silica gel (Biotage, USA), and flash column chromatography was performed on Biotage prepacked columns using the automated flash chromatography system Biotage Isolera One. HPLC purification was performed using Phenomenex Kinetex C18 5 μm 150 x 21.2 mm columns; Eluent A: water + 0.1% trifluoroacetic acid (99%), Eluent B: methanol; DAD scan: (210-400 nm). The 1H- and 13C-NMR spectra were recorded on a Bruker AVANCE 500 MHz spectrometer using deuterated chloroform ($CDCl_3$) as the solvent. Chemical shifts were compared to the residual protonated solvent and are expressed in ppm (δ scale). Peak multiplicities are reported using the following abbreviations: s (singlet), d (doublet), t (triplet), q (quartet), m (multiplet), or br (broad singlet). Mass spectra and purity data were obtained with an Agilent Technologies 6470 series, triple quadrupole LC–MS. The purity of all final tested compounds was determined to be >95% (UV, λ = 254 nm). High-resolution mass analysis was performed using an Agilent 6550 iFunnel Q-TOF mass LC–MS. Compound **99BOC** was prepared according to a previously reported method.[29]

Preparation of 6-(4-amino-4-methylpiperidin-1-yl)-3-(2-chloro-4-(hexyloxy)phenyl)pyrazin-2-amine **(99C6)**.

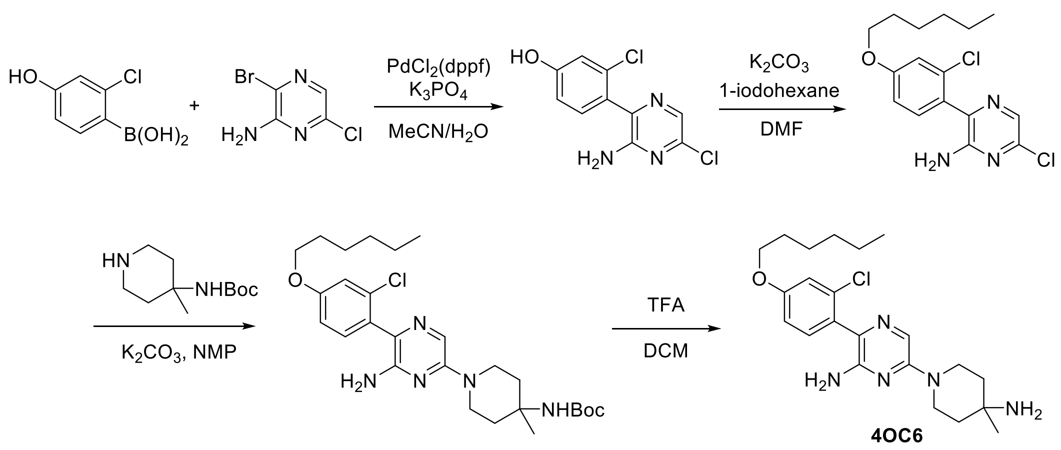

Scheme 1. Synthetic preparation of compound **99C6**.

A mixture of 3-bromo-6-chloropyrazine-2-amine (1.04 g, 5.0 mmol), (2-chloro-4-hydroxyphenyl) boronic acid (0.86 g, 5.0 mmol), $PdCl_2$(dppf)·DCM (204 mg, 0.25 mmol), and potassium phosphate (3.12 g, 15.0 mmol) in MeCN/H_2O (9:1, 10 mL) was stirred for 12 hours at 120°C. After cooling to room temperature, the reaction mixture was filtered through a pad of Celite and then washed with EtOAc. The solvent was removed under reduced pressure, and the resulting residue was purified by silica chromatography (10−50% gradient of EtOAc in hexane) to give 4-(3-amino-5-chloropyrazin-2-yl)-3-chlorophenol (0.89 g, 3.5 mmol) as a yellow solid. MS m/z 256.0 $(M + H)^+$.

1-iodohexane (0.66 mL, 4.5 mmol) was added to the mixture of 4-(3-amino-5-chloropyrazin-2-yl)-3-chlorophenol (0.76 g, 3.0 mmol) and potassium carbonate (0.41 g, 3.0 mmol) in DMF (5 mL). The mixture was then heated and stirred for 6 hours at 60 °C. After cooling to room temperature, the precipitated solid was removed by filtration and washed with ethyl acetate (20 mL). The filtrate was then washed with saturated $NaHCO_3$ aqueous solution and brine. The organic phase was dried over anhydrous Na_2SO_4 and concentrated under vacuum

filtration to obtain the product 6-chloro-3-(2-chloro-4-(hexyloxy)phenyl)pyrazin-2-amine (0.54 g, 1.6 mmol) as a yellow solid. MS m/z 340.1 $(M + H)^+$.

A mixture of 6-chloro-3-(2-chloro-4-(hexyloxy)phenyl)pyrazin-2-amine (340 mg, 1.0 mmol), tert-butyl (4-methylpiperidin-4-yl)carbamate (428 mg, 2.0 mmol), and potassium carbonate (213 mg, 1.0 mmol) in NMP (2 mL) was stirred for 36 hours at 140°C. After cooling to room temperature , the mixture was poured into a separation funnel containing aqueous saturated NH_4Cl and then extracted with EtOAc (3 × 5 mL). The combined organic phases were dried over Na_2SO_4 and vacuum filtered, and the solvents were removed under reduced pressure. The resulting residue was purified by silica chromatography (5−30% gradient of EtOAc in hexane) to give *tert*-butyl (1-(6-amino-5-(2-chloro-4-(hexyloxy)phenyl)pyrazin-2-yl)-4-methylpiperidin-4-yl)carbamate (284 mg, 0.55 mmol) as a yellow solid. MS m/z 518.3 $(M + H)^+$.

A solution of *tert*-butyl (1-(6-amino-5-(2,3-dichlorophenyl)pyrazin-2-yl)-4-methylpiperidin-4-yl)carbamate (130 mg, 0.25 mmol) in DCM (3 mL) was treated with TFA (300 μL), and the resulting mixture was stirred for 6 hours at room temperature. The volatiles were removed under reduced pressure, and the resulting residue was purified with HPLC to afford 6-(4-amino-4-methylpiperidin-1-yl)-3-(2-chloro-4-(hexyloxy)phenyl)pyrazin-2-amine (75 mg, 0.18 mmol) as a yellow solid. 1H-NMR (400 MHz, $CDCl_3$) δ ppm 7.65 – 7.57 (m, 2H), 7.06 (d, J = 2.1 Hz, 1H), 6.92 (dd, J = 9.0, 2.2 Hz, 1H), 6.12 (s, 2H), 3.98 (t, J = 6.5 Hz, 2H), 3.77 (ddd, J = 14.5, 7.1, 4.2 Hz, 2H), 3.62−3.48 (m, 2 H), 1.84 (ddd, J = 13.6, 6.5, 3.8 Hz, 2H), 1.80 – 1.54 (m, 4H), 1.53 – 1.34 (m, 6H), 1.22 (s, 3H), 0.93 – 0.84 (m, 3H). HRMS calcd for $C_{16}H_{20}Cl_2N$ $(M + H)^+$ 418.2368, found 418.2372.

Cloning, expression, and purification of SHP2 protein

The SHP2 protein insert (Aa 1-528) was cloned into a pET-21a(+) vector. Bacterial BL21(DE3) (Novagen) was used as an expression host, and the induction of protein expression was carried out in LB media with 1 mM IPTG at 18°C overnight. Cell pellets were stored at −80°C for subsequent protein purification. Protein purification was conducted at 4°C. Frozen cell pellets were lysed by sonication in 40 ml cold lysis buffer (50 mM Tris-HCl, pH 8.0, 150 mM NaCl, 5 mM imidazole, and 1 mM PMSF) per 1 L of cell pellet. Cell lysates were clarified by centrifugation for 15 min at 6,000 rpm. The supernatant was removed and incubated with HisPur Ni-NTA resin (Thermo Scientific) for 2 h and then packed onto a column and washed with 50 resin volume of Buffer A (50 mM Tris-HCl, pH 8.0, 500 mM NaCl, 5 mM imidazole). The now HIS-tagged proteins were eluted with Buffer B (50 mM Tris-HCl, pH=8.0, 500 mM NaCl, 300 mM imidazole,). Pooled HIS-protein-containing fractions were concentrated, loaded onto a HiLoad 26/600 Superdex 75 column (GE Healthcare Biosciences), and eluted with storage buffer (50 mM Tris-HCl, pH 8.0, 150 mM NaCl, 1 mM DTT, 10% glycerol). Proteins used for inhibition assays were purified using Ni-NTA resin (Qiagen) followed by size exclusion column chromatography (ÄKTA pure, Cytiva) and the purity was determined to be >95% by SDS-PAGE and Coomassie staining. The protein was aliquoted and stored at −80°C.

SHP2 allosteric inhibition assay

Catalytic activity of SHP2 (1-528) was assayed with 6,8-Difluoro-4-Methylumbelliferyl Phosphate (DiFMUP, Invitrogen, cat# D6567) as a substrate in 3,3-Dimethylglutaric acid (DMG) buffer (50 mM DMG, pH 7.0, 1 mM EDTA, 18 mM NaCl, 0.01% Triton X-100) at

25°C. To determine the IC_{50} values, the assays were performed in 96-well plates (Corning Costar 3915). Serially diluted compounds were incubated with 0.5 nM of SHP2 and 0.5 μM of peptide IRS1_pY1172(dPEG8)pY1222 (sequence H2N-LN(pY)IDLDLV(dPEG8)LST(pY)ASINFQK-amide) for 30 min at room temperature. The substrate DiFMUP was then added to the reaction (200 μM final concentration, total reaction volume is 200 μL) and incubated at 25°C for 10 min. The reaction was then quenched by adding 40 μL of 160 μM bpV(Phen) (Sigma-Aldrich, cat# SML0889) solution. The fluorescence signal was monitored with a CLARIOstar Plus Microplate Spectrophotometer (BMG Labtech) using excitation and emission wavelengths of 340 and 450 nm, respectively. The dose-response curves of the SHP2-AIs were analyzed using normalized IC_{50} regression curve fitting with control-based normalization. Data were fitted using Prism GraphPad 9.2.0.

Supplementary Figures

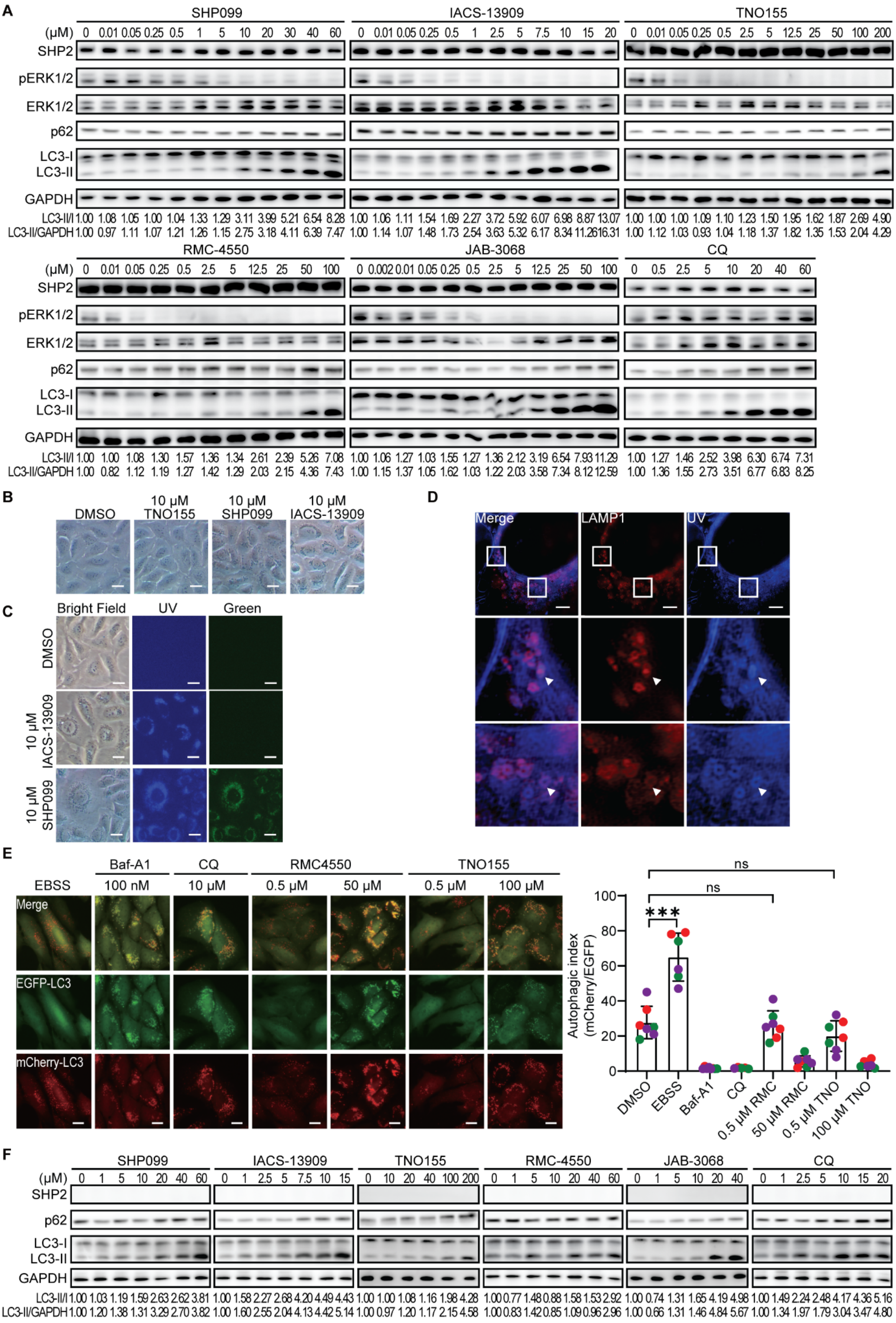
A
SHP099
IACS-13909
TNO155
(μM) 0 0.01 0.05 0.25 0.5 1 5 10 20 30 40 60
0 0.01 0.05 0.25 0.5 1 2.5 5 7.5 10 15 20
0 0.01 0.05 0.25 0.5 2.5 5 12.5 25 50 100 200
SHP2
pERK1/2
ERK1/2
p62
LC3-I
LC3-II
GAPDH
LC3-II/I 1.00 1.08 1.05 1.00 1.04 1.33 1.29 3.11 3.99 5.21 6.54 8.28 1.00 1.06 1.11 1.54 1.69 2.27 3.72 5.92 6.07 6.98 8.87 13.07 1.00 1.00 1.00 1.09 1.10 1.23 1.50 1.95 1.62 1.87 2.69 4.90
LC3-II/GAPDH 1.00 0.97 1.11 1.07 1.21 1.26 1.15 2.75 3.18 4.11 6.39 7.47 1.00 1.14 1.07 1.48 1.73 2.54 3.63 5.32 6.17 8.34 11.26 16.31 1.00 1.12 1.03 0.93 1.04 1.18 1.37 1.82 1.35 1.53 2.04 4.29
RMC-4550
JAB-3068
CQ
(μM) 0 0.01 0.05 0.25 0.5 2.5 5 12.5 25 50 100
0 0.002 0.01 0.05 0.25 0.5 2.5 5 12.5 25 50 100
0 0.5 2.5 5 10 20 40 60
LC3-II/I 1.00 1.00 1.08 1.30 1.57 1.36 1.34 2.61 2.39 5.26 7.08 1.00 1.06 1.27 1.03 1.55 1.27 1.36 2.12 3.19 6.54 7.93 11.29 1.00 1.27 1.46 2.52 3.98 6.30 6.74 7.31
LC3-II/GAPDH 1.00 0.82 1.12 1.19 1.27 1.42 1.29 2.03 2.15 4.36 7.43 1.00 1.15 1.37 1.05 1.62 1.03 1.22 2.03 3.58 7.34 8.12 12.59 1.00 1.36 1.55 2.73 3.51 6.77 6.83 8.25
B
DMSO
10 μM TNO155
10 μM SHP099
10 μM IACS-13909
C
Bright Field
UV
Green
DMSO
10 μM IACS-13909
10 μM SHP099
D
Merge
LAMP1
UV
E
EBSS
Baf-A1 100 nM
CQ 10 μM
RMC4550 0.5 μM 50 μM
TNO155 0.5 μM 100 μM
Merge
EGFP-LC3
mCherry-LC3
Autophagic index (mCherry/EGFP)
ns
ns
***
DMSO
EBSS
Baf-A1
CQ
0.5 μM RMC
50 μM RMC
0.5 μM TNO
100 μM TNO
F
SHP099
IACS-13909
TNO155
RMC-4550
JAB-3068
CQ
(μM) 0 1 5 10 20 40 60
0 1 2.5 5 7.5 10 15
0 10 20 40 100 200
0 1 5 10 20 40 60
0 1 5 10 20 40
0 1 2.5 5 10 15 20
SHP2
p62
LC3-I
LC3-II
GAPDH
LC3-II/I 1.00 1.03 1.19 1.59 2.63 2.62 3.81 1.00 1.58 2.27 2.68 4.20 4.49 4.43 1.00 1.00 1.08 1.16 1.98 4.28 1.00 0.77 1.48 0.88 1.58 1.53 2.92 1.00 0.74 1.31 1.65 4.19 4.98 1.00 1.49 2.24 2.48 4.17 4.36 5.16
LC3-II/GAPDH 1.00 1.20 1.38 1.31 3.29 2.70 3.82 1.00 1.60 2.55 2.04 4.13 4.42 5.14 1.00 0.97 1.20 1.17 2.15 4.58 1.00 0.83 1.42 0.85 1.09 0.96 2.96 1.00 0.66 1.31 1.46 4.84 5.67 1.00 1.34 1.97 1.79 3.04 3.47 4.80

Supplemental Figure 1. SHP2 allosteric inhibitors have SHP2-independent lysosomal membrane localization and autophagy inhibition. (**A**) WT HEK293 cells were treated with a series of concentrations of SHP099, TNO155, IACS-13909, RMC-4550, or JAB-3068 for 6 hours. Total lysates were used for immunoblots. (**B**) U2OS cells were treated with DMSO, 10 μM SHP099, TNO155 or IACS-13909 for 6 hours. Live cell imaging shows accumulation of cytoplasmic vacuolization after SHP099 and IACS-13909 treatment. Scale bar, 10 μm. (**C**) U2OS cells were treated with DMSO, 10 μM SHP099 (green and blue), or IACS-13909 (blue) for 6 hours. Live cell imaging shows subcellular localization of the compounds. Scale bar, 10 μm. (**D**) U2OS cells were treated with 5 μM IACS-13909 (blue) for 6 hours. Cells were fixed and stained with LAMP1 (red) antibody for structured illumination microscopy. Scale bar, 2 μm. (**E**) EGFP-mCherry-LC3 expressing U2OS cells were treated with EBSS or indicated compounds for 6 hours. Representative images of EGFP (green, pH sensitive), mCherry (red, pH insensitive), and merged channels were displayed. Scale bar, 10 μm. Autophagic index indicates the ratio of the areas of mCherry+ punctae to EGFP+ punctae. Mean autophagic index is plotted, with each individual data point representing one analyzed cell fields (5-10 fields total) from 3 independent experiments (labeled with different colors). Data are represented as means ± SD. Significance determined by one-way ANOVA followed by Dunnett multiple comparison test.(**F**) SHP2 KOHEK293 cells were treated with a series of concentrations of SHP099, TNO155, IACS-13909, RMC-4550, or JAB-3068 for 6 hours. Total lysates were used for immunoblots. Representative data from 3 independent experiments displayed for all panels.

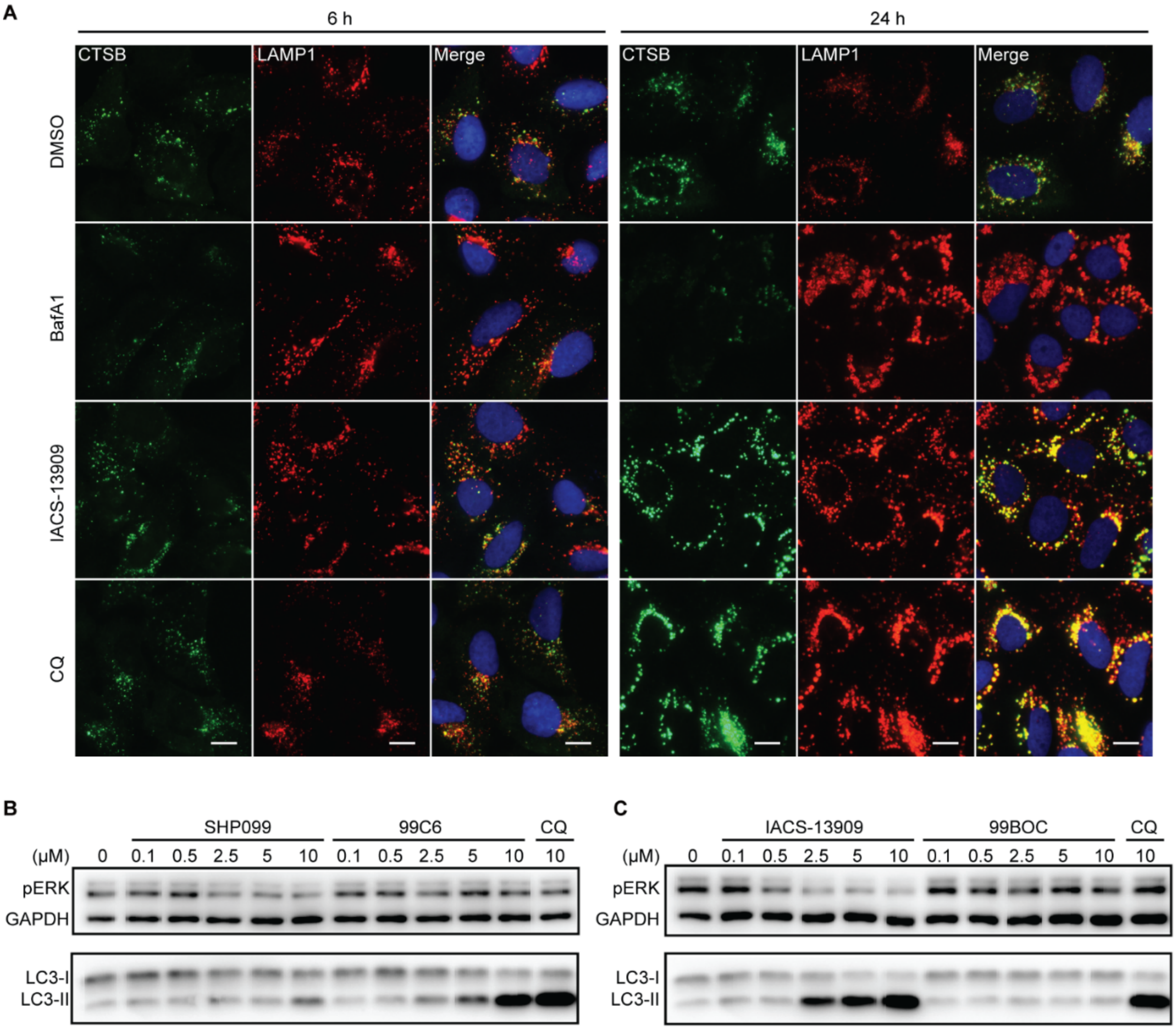

Supplemental Figure 2. The cationic amphiphilic feature of SHP2 allosteric inhibitors creates lysosomal dysfunction and autophagy inhibition. (**A**) U2OS cells were treated with DMSO, 100 nM BafA1, 5 µM IACS-13909, or 10 µM CQ for 6 or 24 hours. Cells were fixed and stained with CTSB (green) and LAMP1 (red) antibody for confocal imaging. Scale bar, 10 µm. (**B**) and (**C**) HEK293 cells were treated with a series of doses of SHP099, 99C6, IACS-13909, or 99BOC for 6 hours. Total lysates were used for immunoblots. Representative data from 3 independent experiments displayed for all panels.

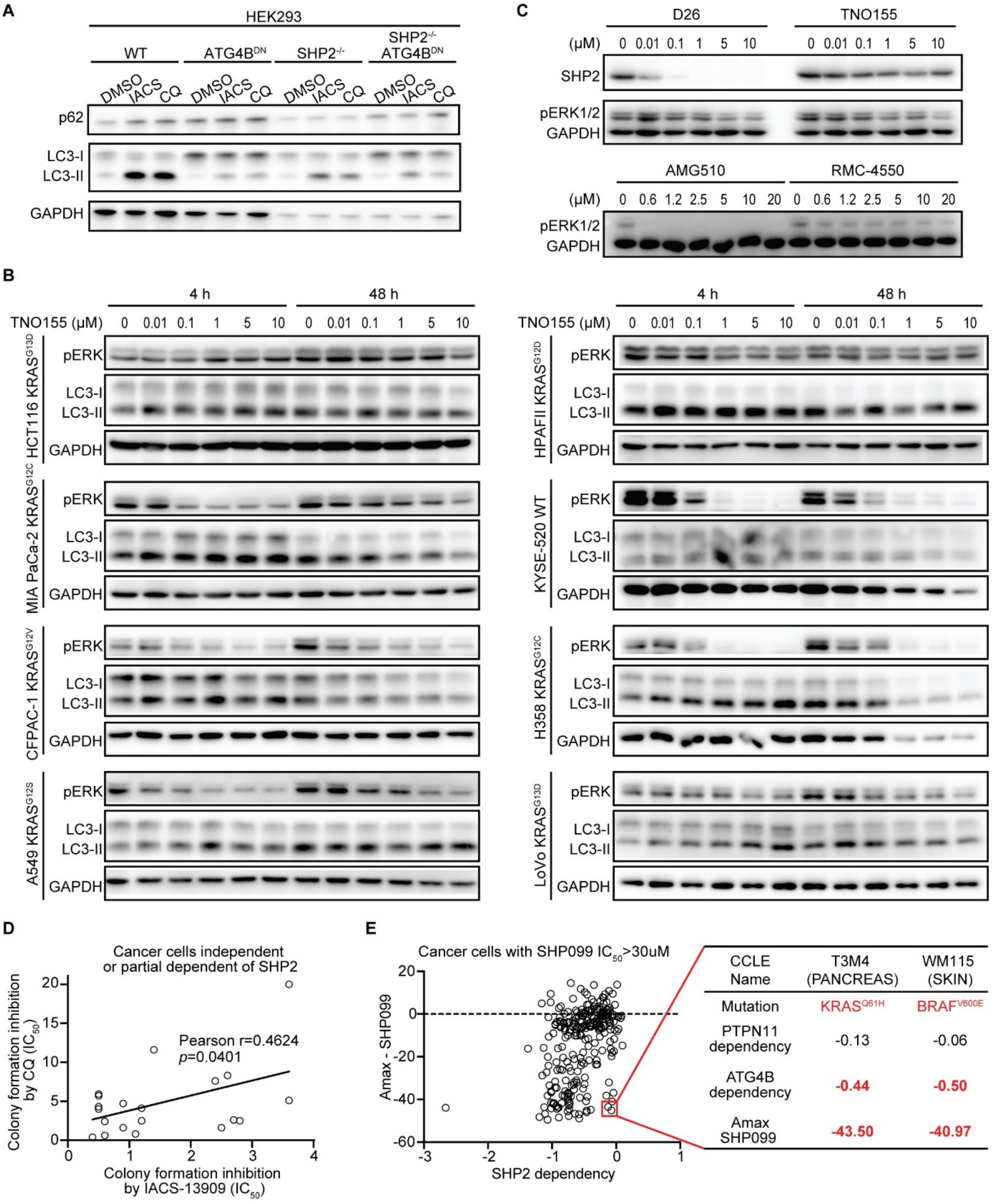

Supplemental Figure 3. Differentiating the mode-of-action of SHP2 allosteric inhibitors through SHP2/ATG4B dependency. (**A**) WT or SHP2$^{-/-}$ HEK293 cells with or without overexpression of the dominant negative ATG4B^{C74A} mutation were treated with DMSO, 5 μM IACS-13909, or 10 μM CQ for 6 hours. Total lysates were used for immunoblots. ATG4B^{C74A} overexpressing cells show deficient autophagosome formation and low LC3-II levels. (**B**) A

panel of 8 cancer cell lines was treated with a series of TNO155 concentrations for 4 or 48 hours. Cell-line-dependent changes in pERK show that RAS status is correlated with differential dependencies on SHP2. Total lysates were used for immunoblots. (**C**) Mia-PaCa2 cells were treated with a series of concentrations of D26, TNO155, AMG510 or RMC-4550 for 6 hours. Total lysates were used for immunoblots. (**D**) Correlation of the IC_{50} values of CQ and IACS-13909 determined by the colony formation assay. (**E**) Correlation of SHP2 and ATG4B dependency with the effectiveness of SHP099 in SHP2-independent cell lines in the Depmap database. Representative data from 3 independent experiments displayed for panel A and C. Panel B is the Representative data from 2 independent experiments.

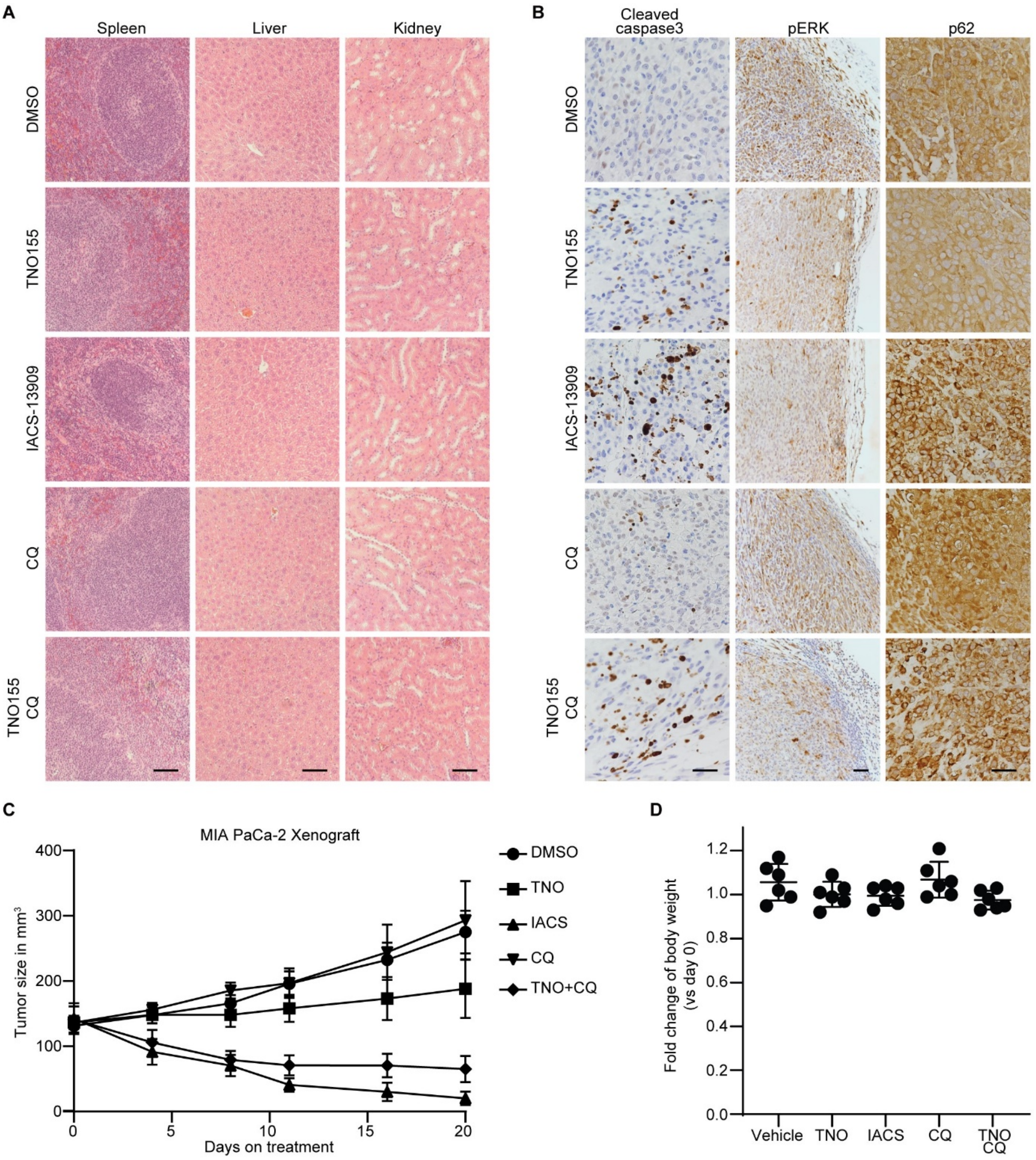

Supplemental Figure 4. In vivo activity of SHP2 single inhibition vs SHP2/autophagy dual inhibition. (**A**) Representative (from 5 individual organs) images of H&E staining of mouse organs after treatment with vehicle control, 50 mg/kg TNO155, 50 mg/kg IACS-13909, 50 mg/kg CQ, or CQ plus TNO155. Scale bar, 100 µm. (**B**) Representative (from 5 individual tumors) immunohistochemistry images of tumors from MiaPaCa-2 xenograft-bearing mice treated with vehicle control, 50 mg/kg TNO155, 50 mg/kg IACS-13909, 50 mg/kg CQ, or CQ plus TNO155. Scale bar, 100 µm. (**C**) MIA PaCa-2 tumor growth curve of

after treatment with vehicle control, 50 mg/kg TNO155, 50 mg/kg IACS-13909, 50 mg/kg CQ, or CQ plus TNO155. (Mean±SEM, n=6-10/treatment group). (**D**) Body weight changes in MiaPaCa-2 xenograft-bearing mice after treatment with vehicle control, 50 mg/kg TNO155, 50 mg/kg IACS-13909, 50 mg/kg CQ, or CQ plus TNO155.

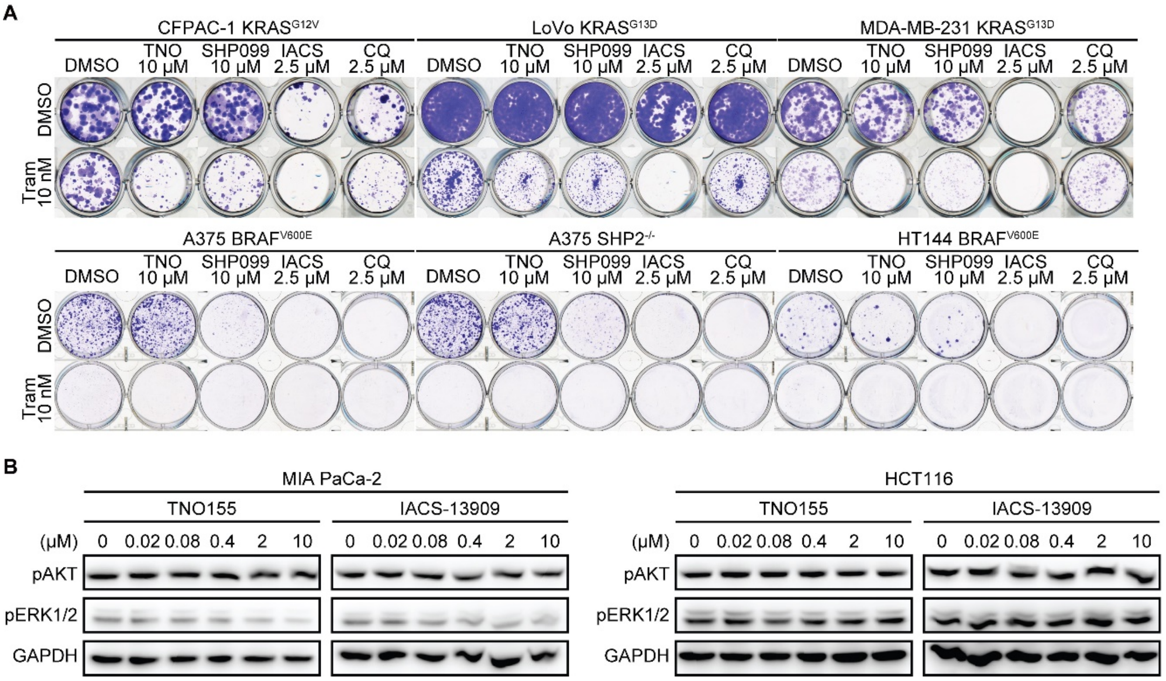

Supplemental Figure 5. MEK/SHP2/autophagy triple inhibition is highly effective in KRAS-mutated cancer cells. (**A**) Results of colony formation assays using a panel of cancer cell lines treated with DMSO or indicated compounds for 10 days. (**B**) MiaPaCa-2 and H116 cells were treated with a series of concentrations of TNO155 or IACS-13909 for 6 hours. Decreases in pERK but not pAKT levels are shown. Total lysates were used for immunoblots. Representative data from 3 independent experiments displayed for panel A and B.

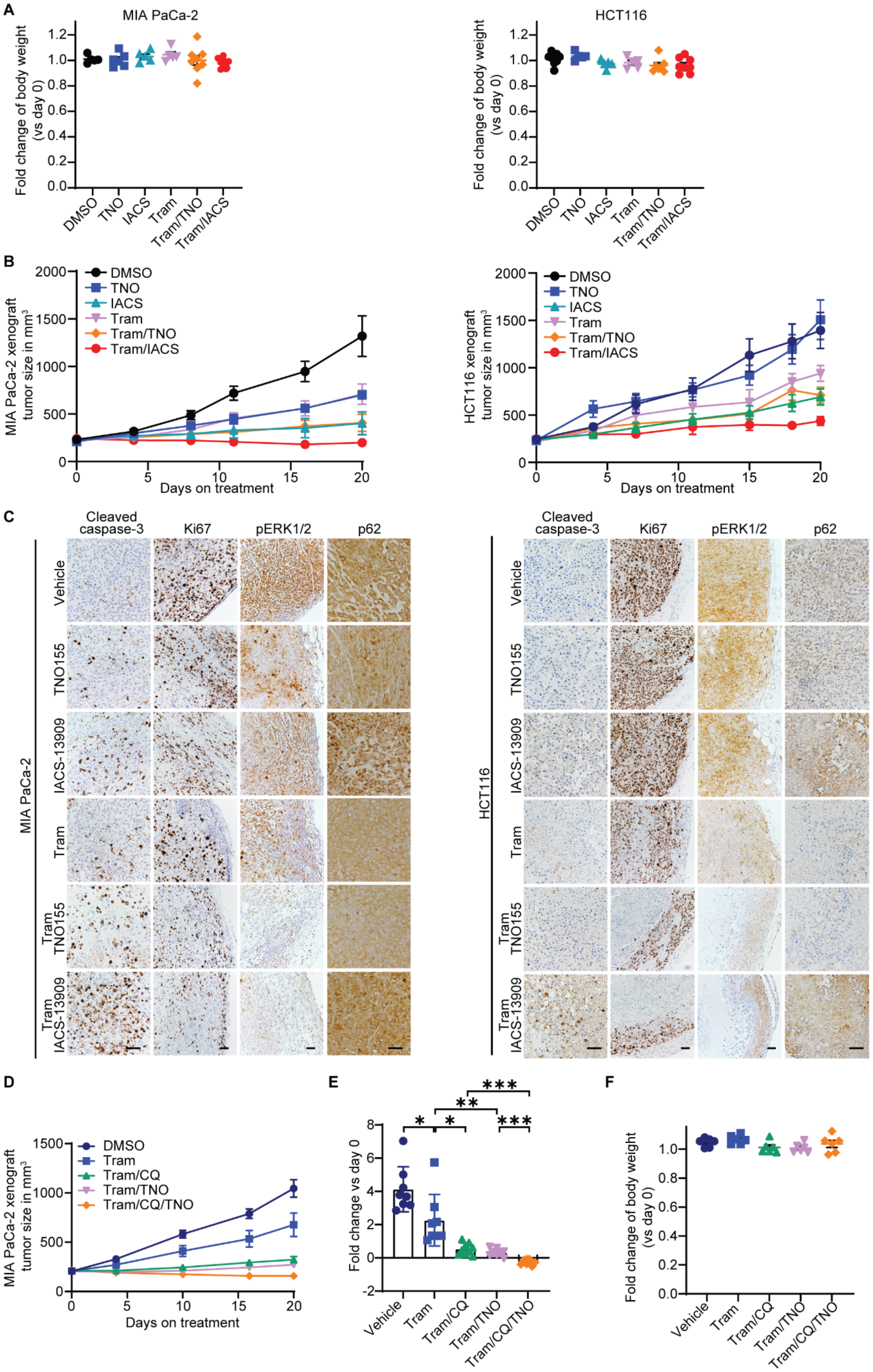
A
MIA PaCa-2
HCT116
Fold change of body weight (vs day 0)
DMSO
TNO
IACS
Tram
Tram/TNO
Tram/IACS
B
MIA PaCa-2 xenograft tumor size in mm³
HCT116 xenograft tumor size in mm³
Days on treatment
C
Cleaved caspase-3
Ki67
pERK1/2
p62
Vehicle
TNO155
IACS-13909
Tram
Tram TNO155
Tram IACS-13909
MIA PaCa-2
HCT116
D
MIA PaCa-2 xenograft tumor size in mm³
DMSO
Tram
Tram/CQ
Tram/TNO
Tram/CQ/TNO
Days on treatment
E
Fold change vs day 0
Vehicle
Tram
Tram/CQ
Tram/TNO
Tram/CQ/TNO
F
Fold change of body weight (vs day 0)
Vehicle
Tram
Tram/CQ
Tram/TNO
Tram/CQ/TNO

Supplemental Figure 6. MEK/SHP2/autophagy triple inhibition is highly effective in KRAS-mutated tumors in vivo. (**A**) Body weight changes of MiaPaCa-2 or HCT 116 xenograft-bearing mice after treatment with vehicle control, 40 mg/kg TNO155, 40 mg/kg IACS-13909, 0.25 mg/kg Trametinib, TNO155 plus Trametinib, or IACS-13909 plus Trametinib. (**B**) MIA PaCa-2 tumor growth curve of after treatment with indicated compounds. (Mean±SEM, n=5-10/treatment group). (**C**) Representative (from 5 individual tumors) immunohistochemistry images of Mia-PaCa2 and H116 xenografts after treatment with indicated compounds. Scale bar, 100 µm. (**D-F**) MIA PaCa-2 tumor growth curve, endpoint tumor volume change, and body weight change of after treatment with vehicle control, 0.25 mg/kg Trametinib, Trametinib plus 40 mg/kg TNO155, Trametinib plus 50 mg/kg Chloroquine, or Trametinib plus Chloroquine and TNO155. Significance was determined by Brown-Forsythe and Welch ANOVA test followed by Two-stage linear step-up procedure of Benjamini, Krieger and Yekutieli. (Mean±SEM for growth curve, means ± SD for the endpoint and body weight change. n=5-10/treatment group)
