# Supplementary material for: Off-target autophagy inhibition by SHP2 allosteric inhibitors contributes to their antitumor activity in RAS-driven cancers
Source: J Clin Invest. 2024 Jun 6;134(15):e177142. doi: 10.1172/JCI177142 (PMC11291269; doi:10.1172/JCI177142)

Full unedited gel for Figure 2C

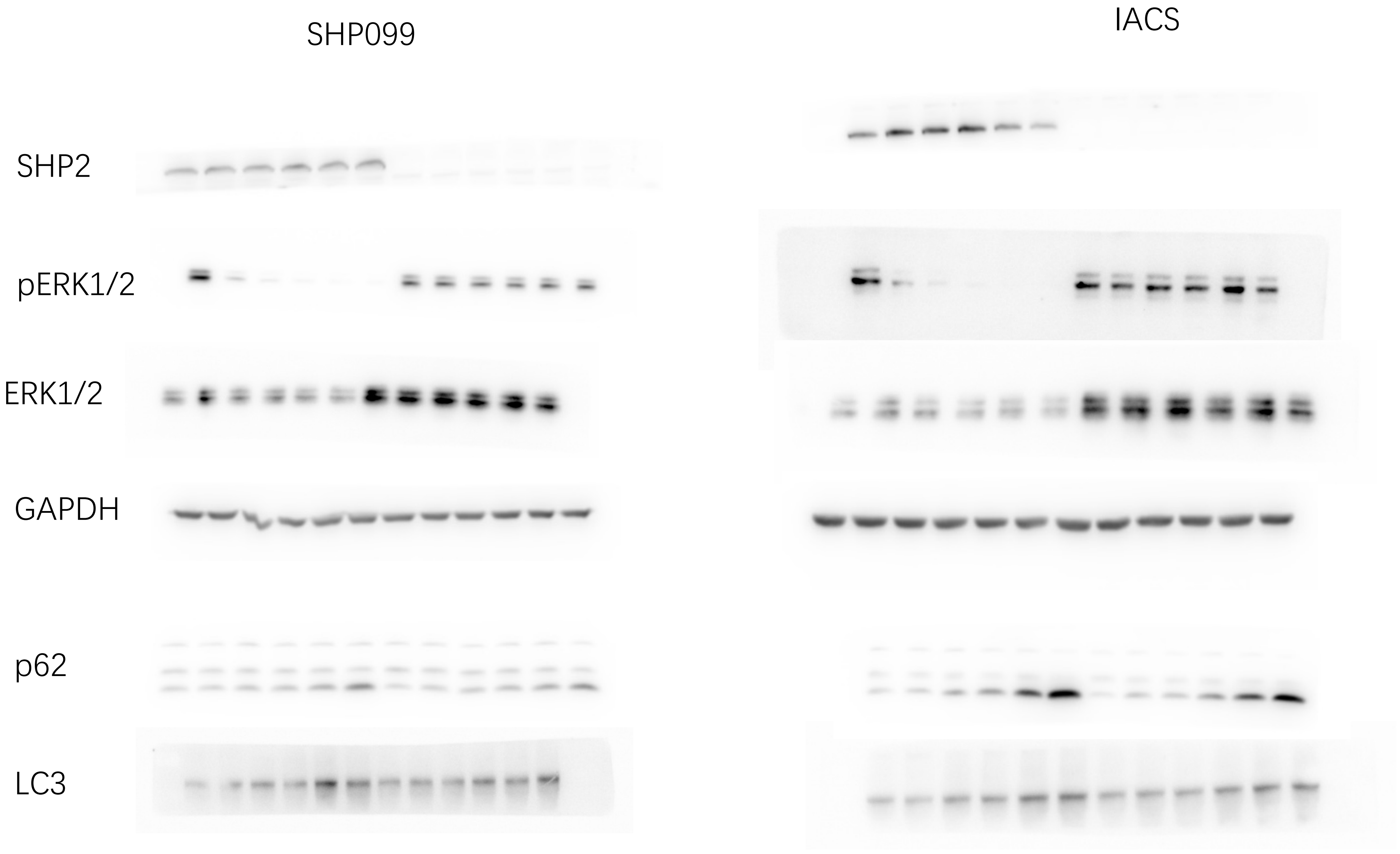

Full unedited gel for Figure 2D

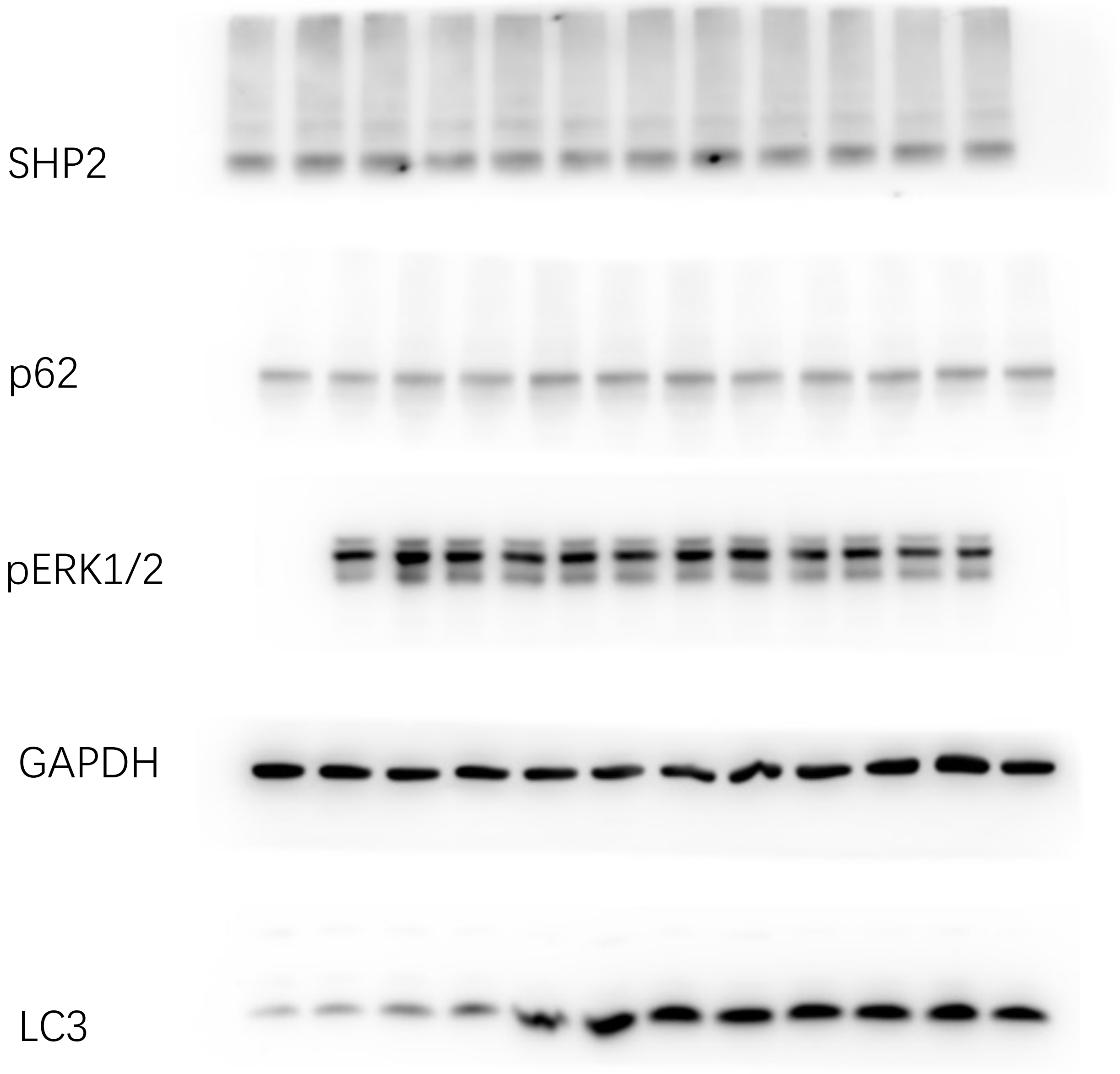

Full unedited gel for Figure 2F

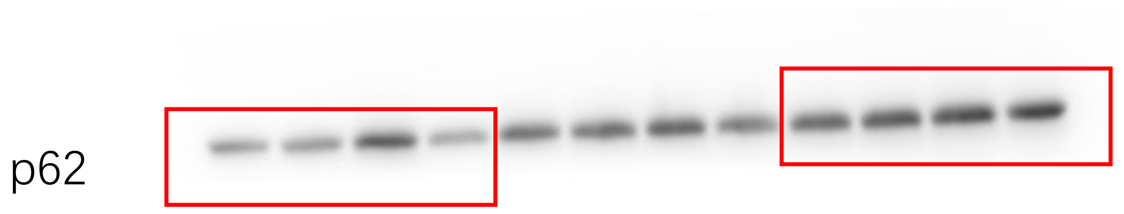

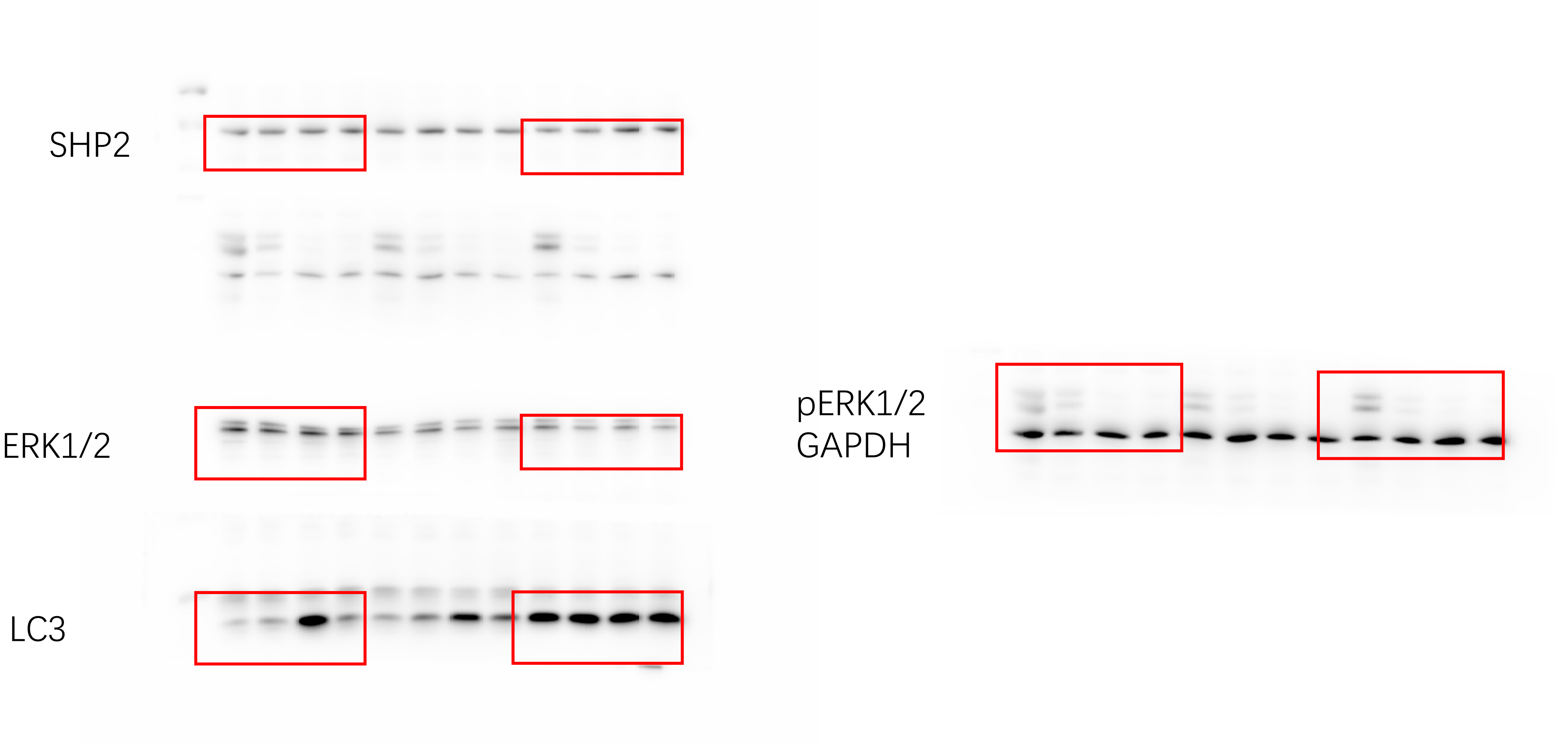

Full unedited gel for Figure 4B

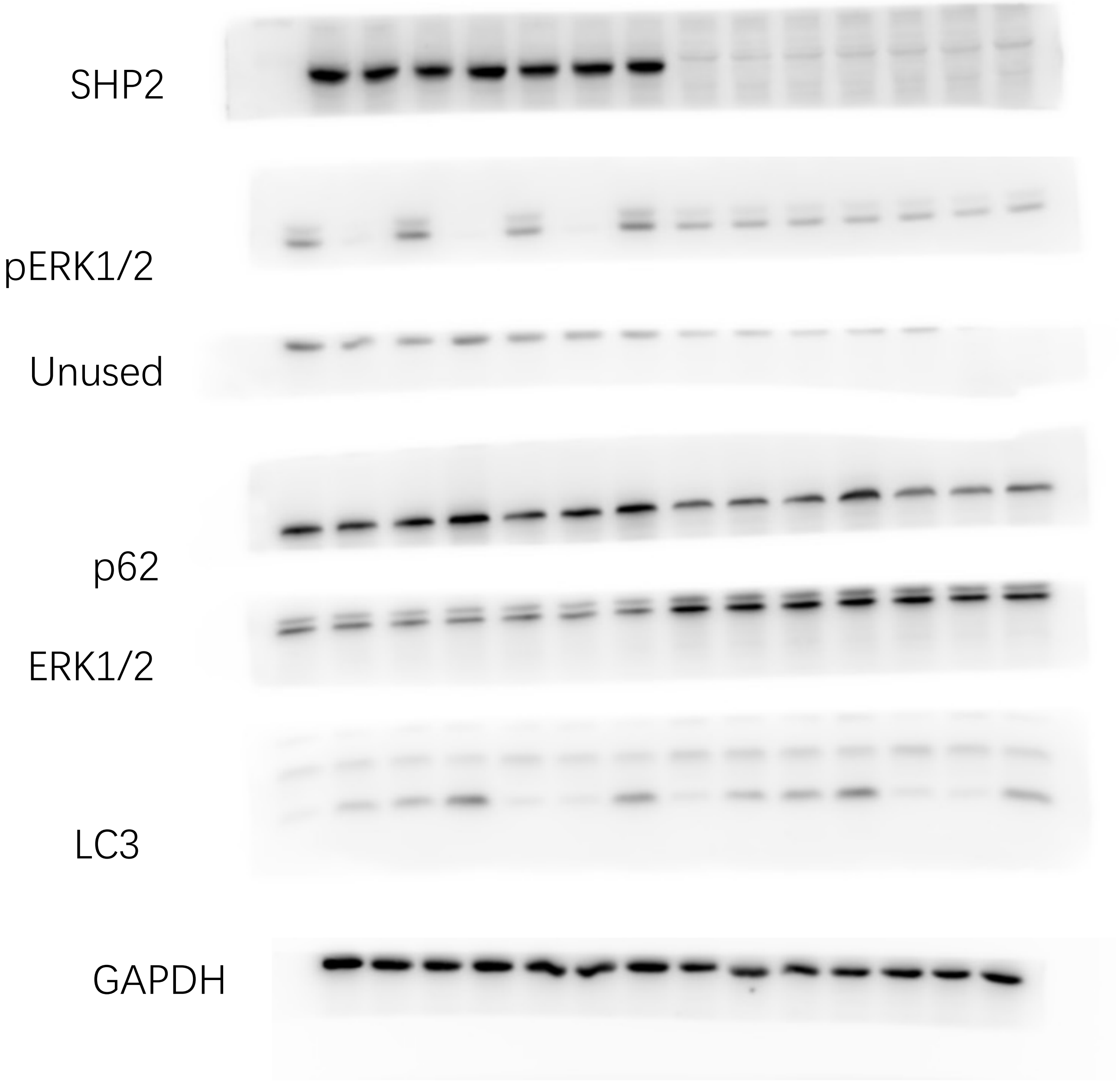

Full unedited gel for Figure 6B&C

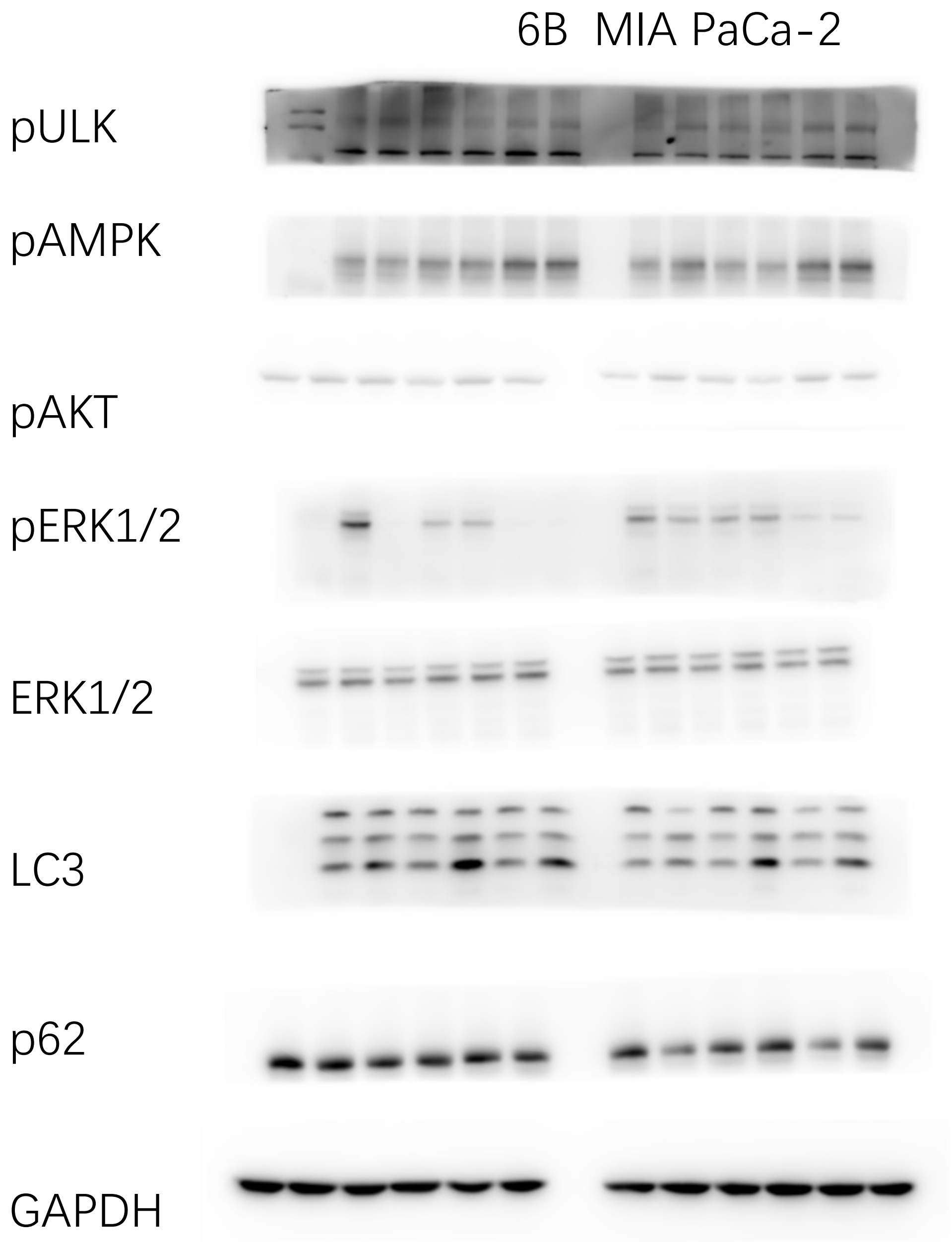

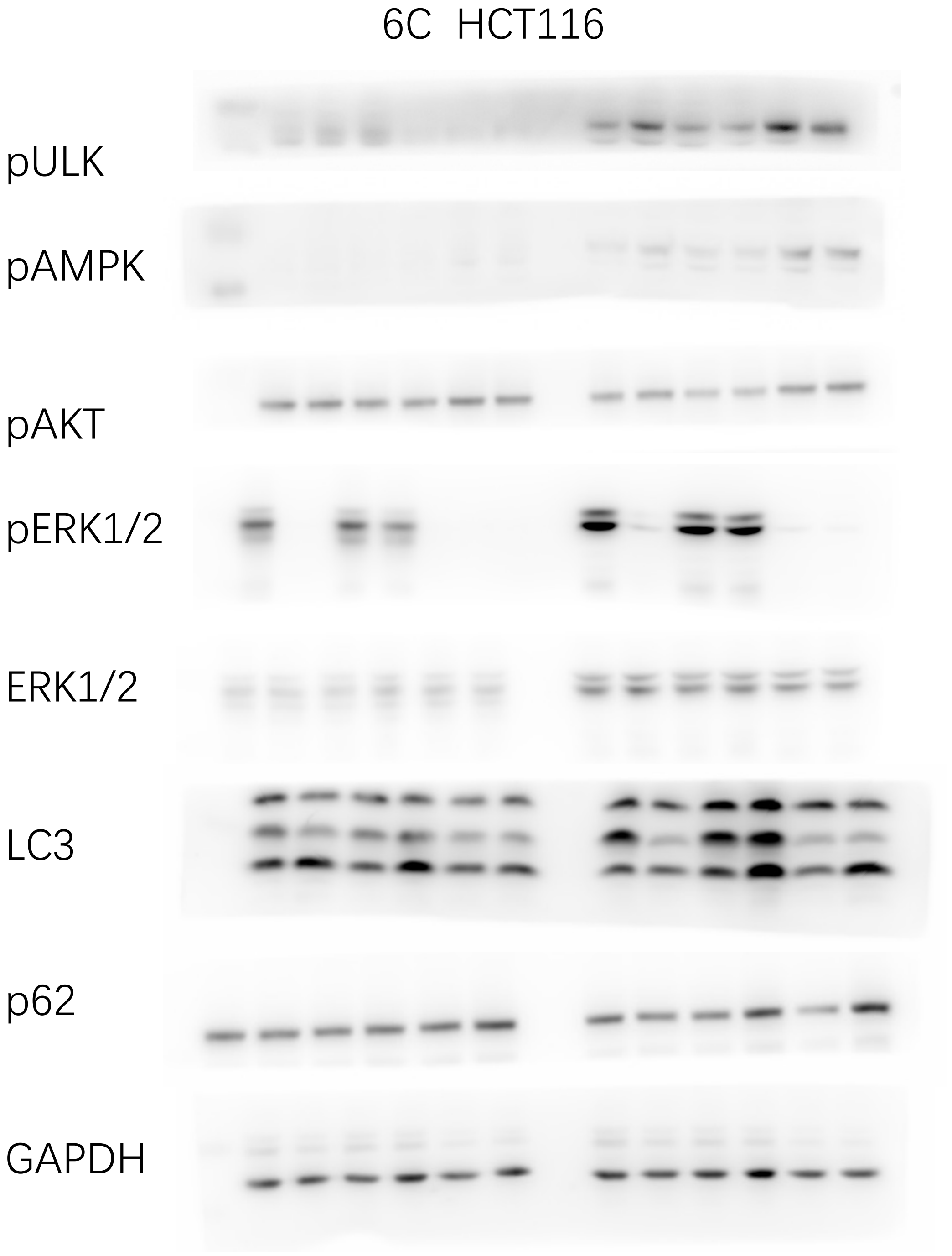

Full unedited gel for Figure 6D&E

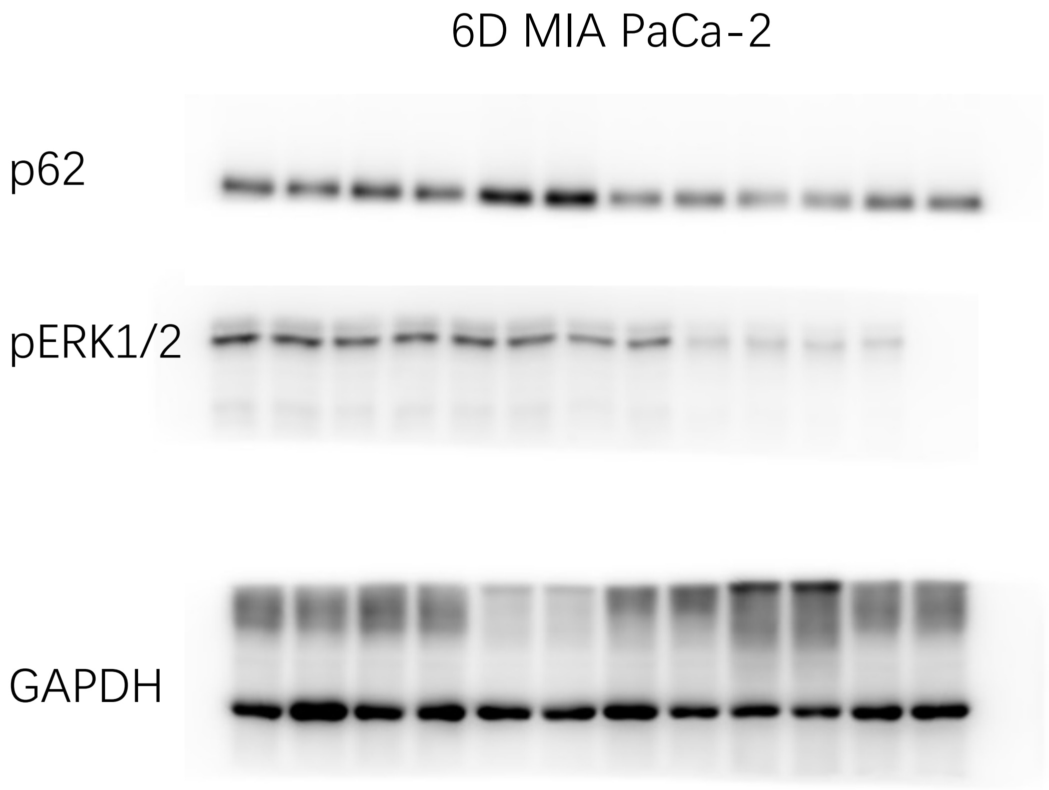

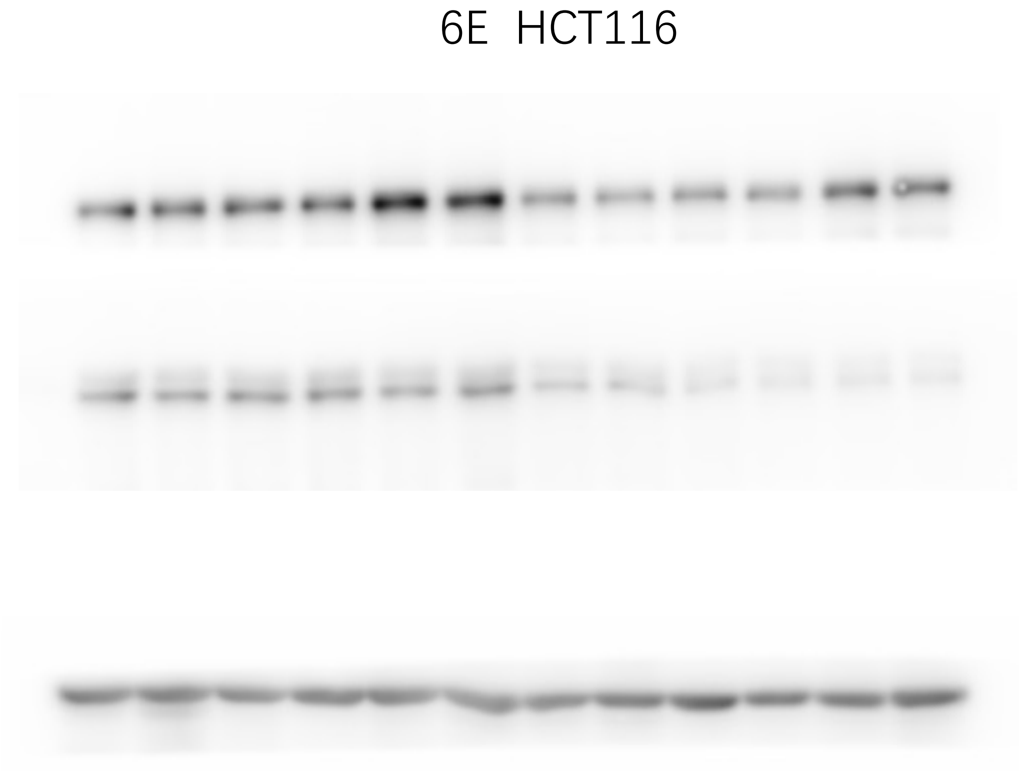

Full unedited gel for Supplemental Figure 1A

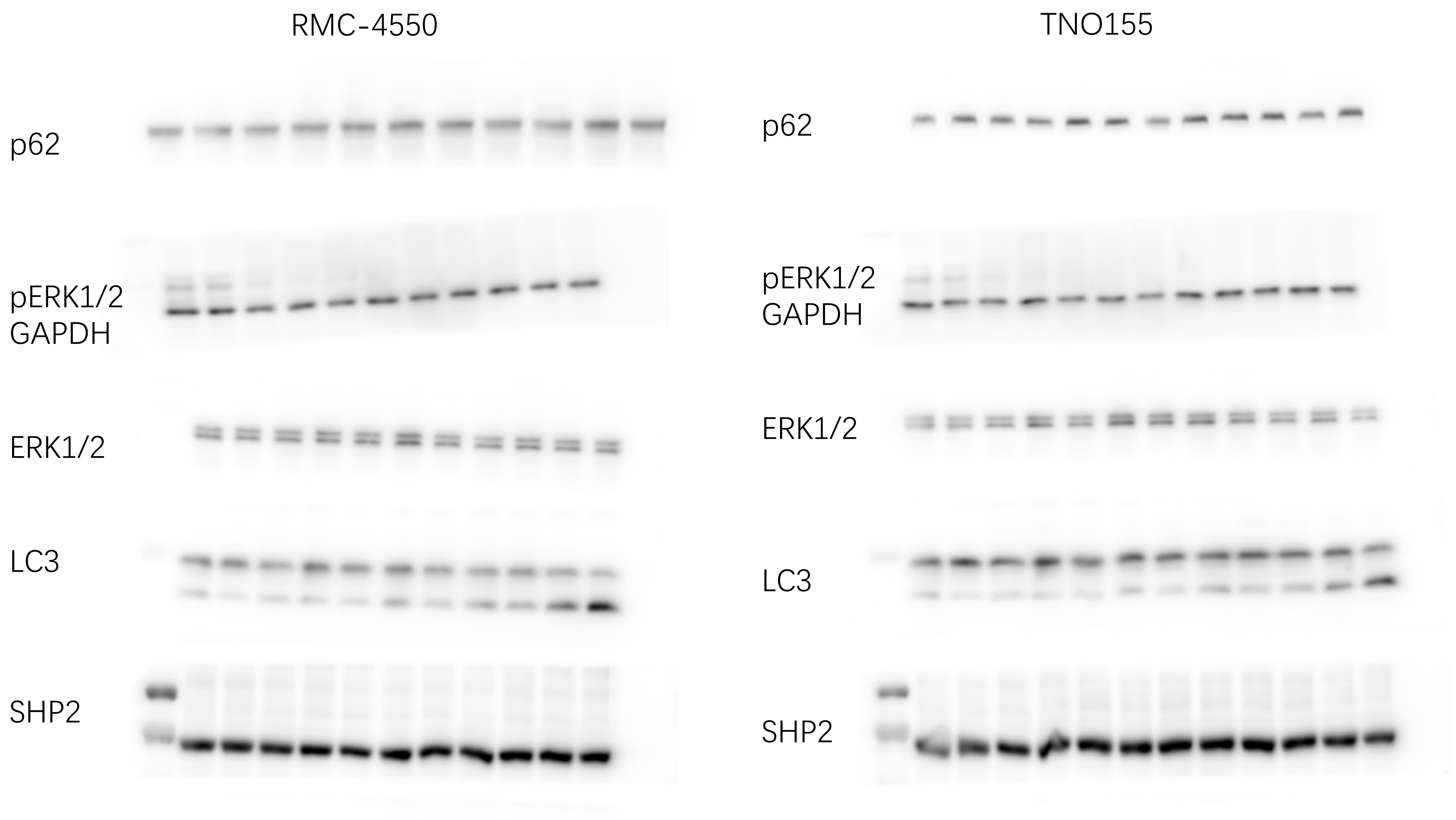

Full unedited gel for Supplemental Figure S1A

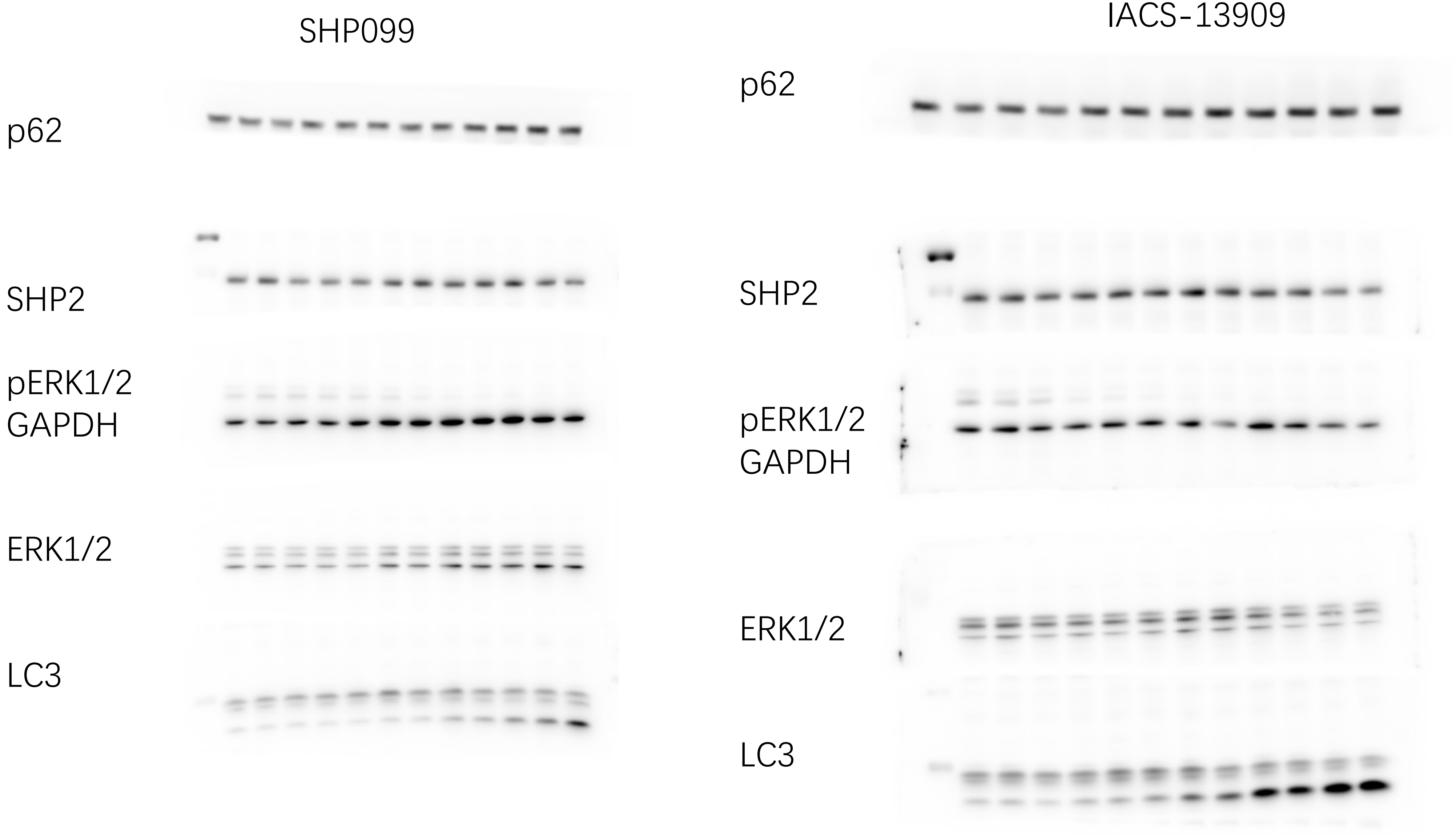

Full unedited gel for Supplemental Figure S1A

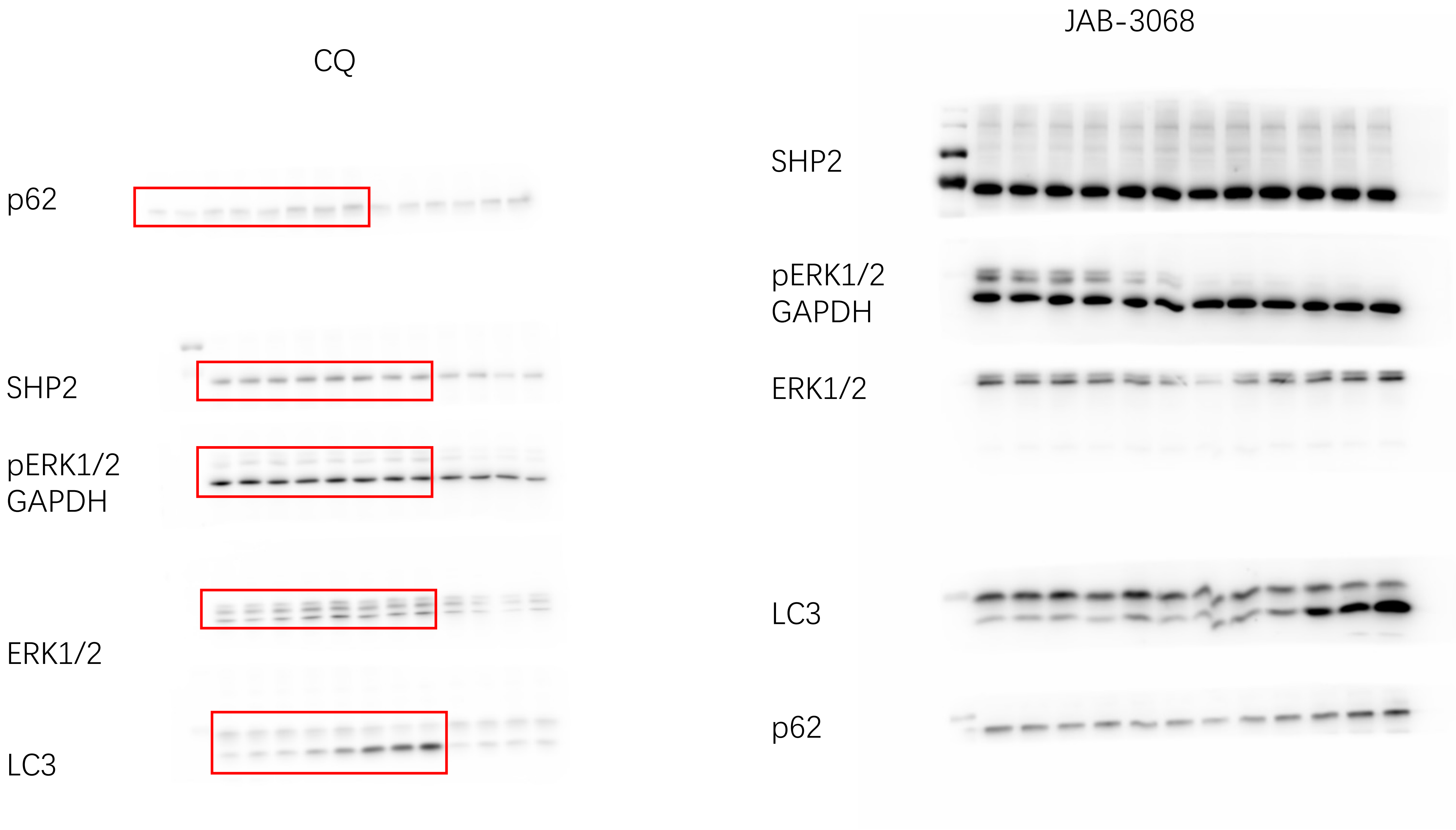

Full unedited gel for Supplemental Figure S1F

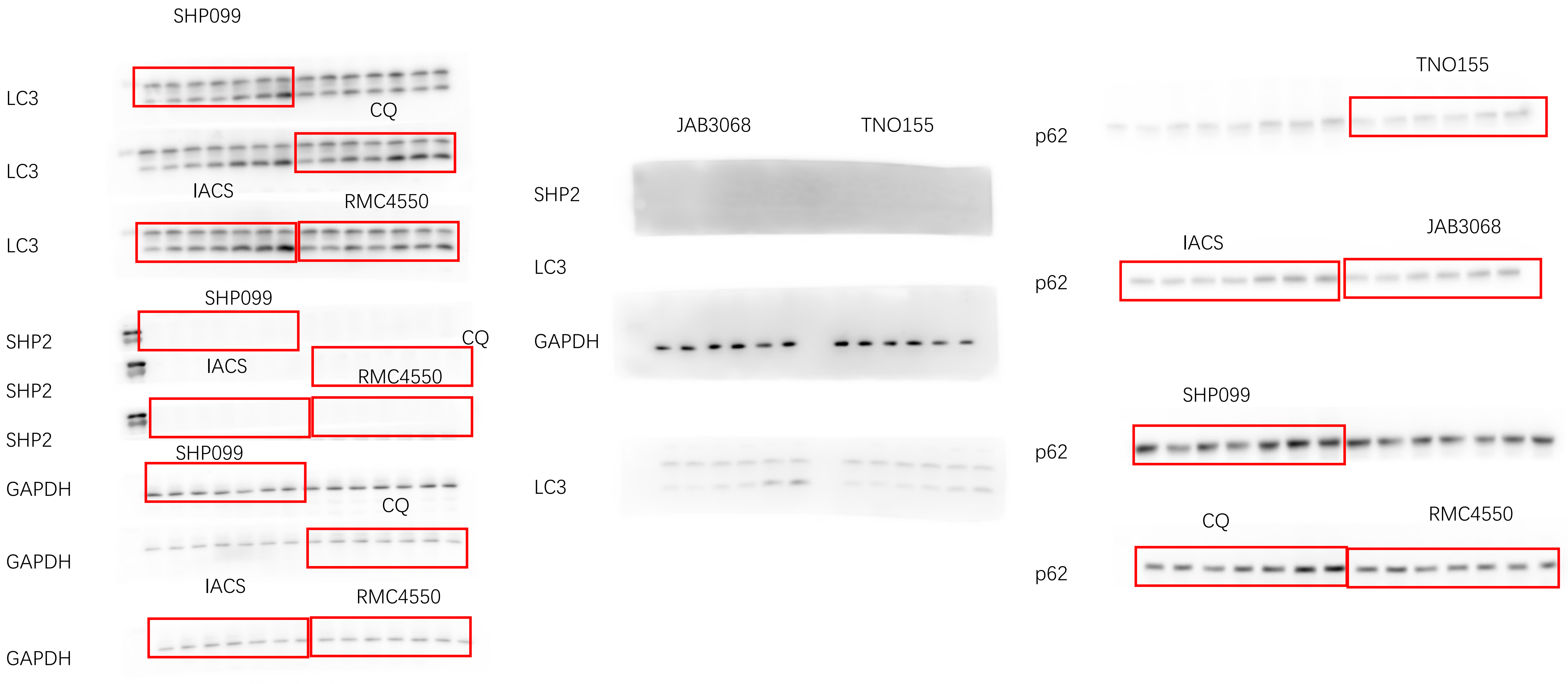

Full unedited gel for Supplemental Figure S2B

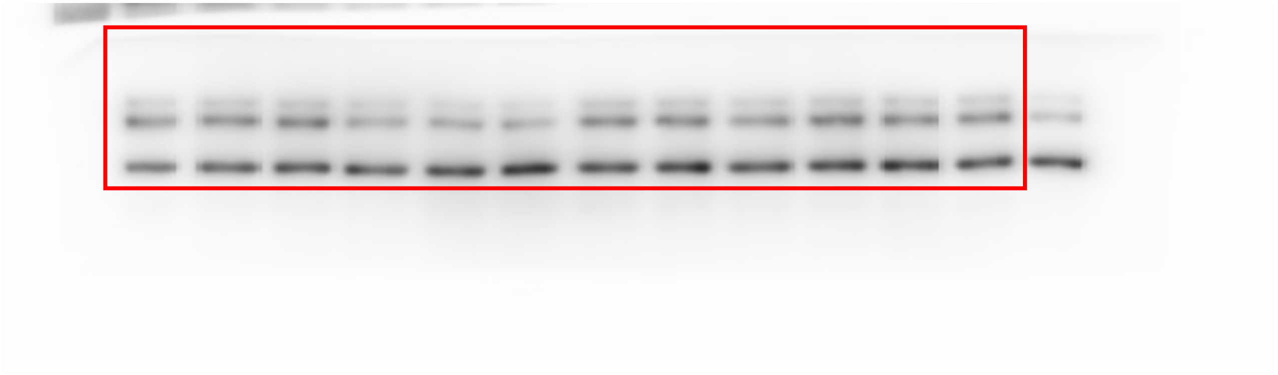

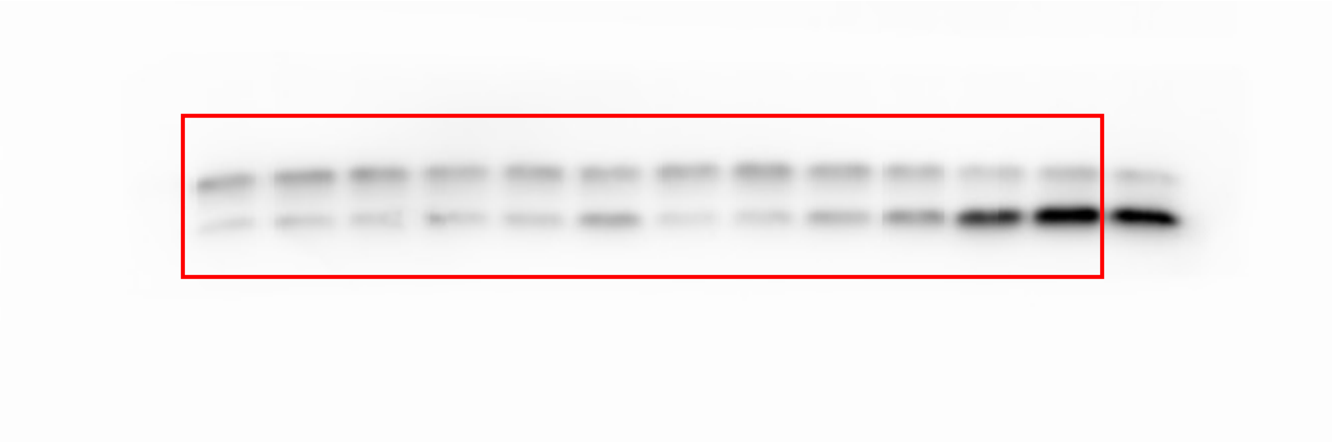

Full unedited gel for Supplemental Figure S2C

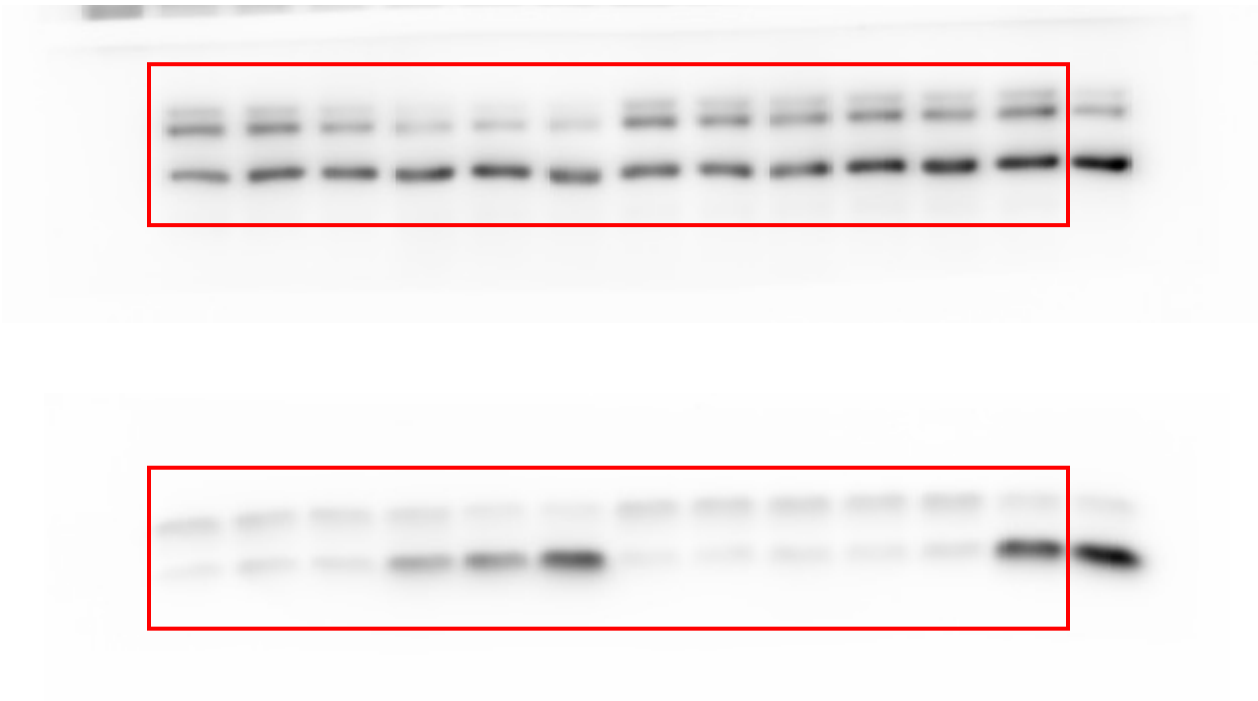

Full unedited gel for Supplemental Figure S3A

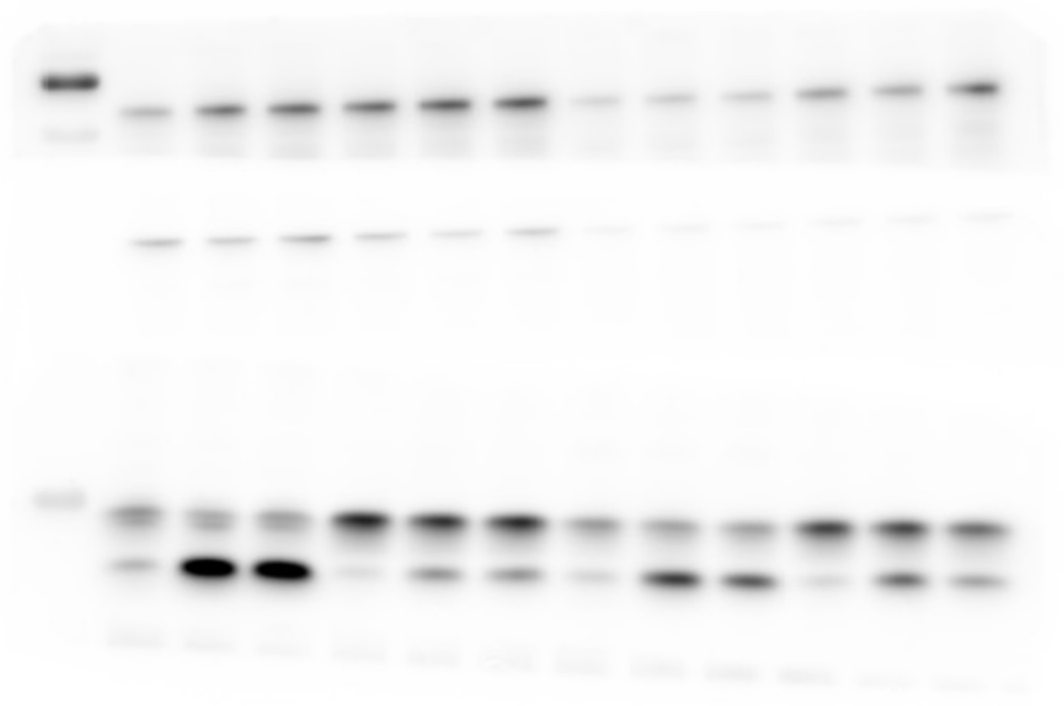

Full unedited gel for Supplemental Figure S3B

pERK and GAPDH

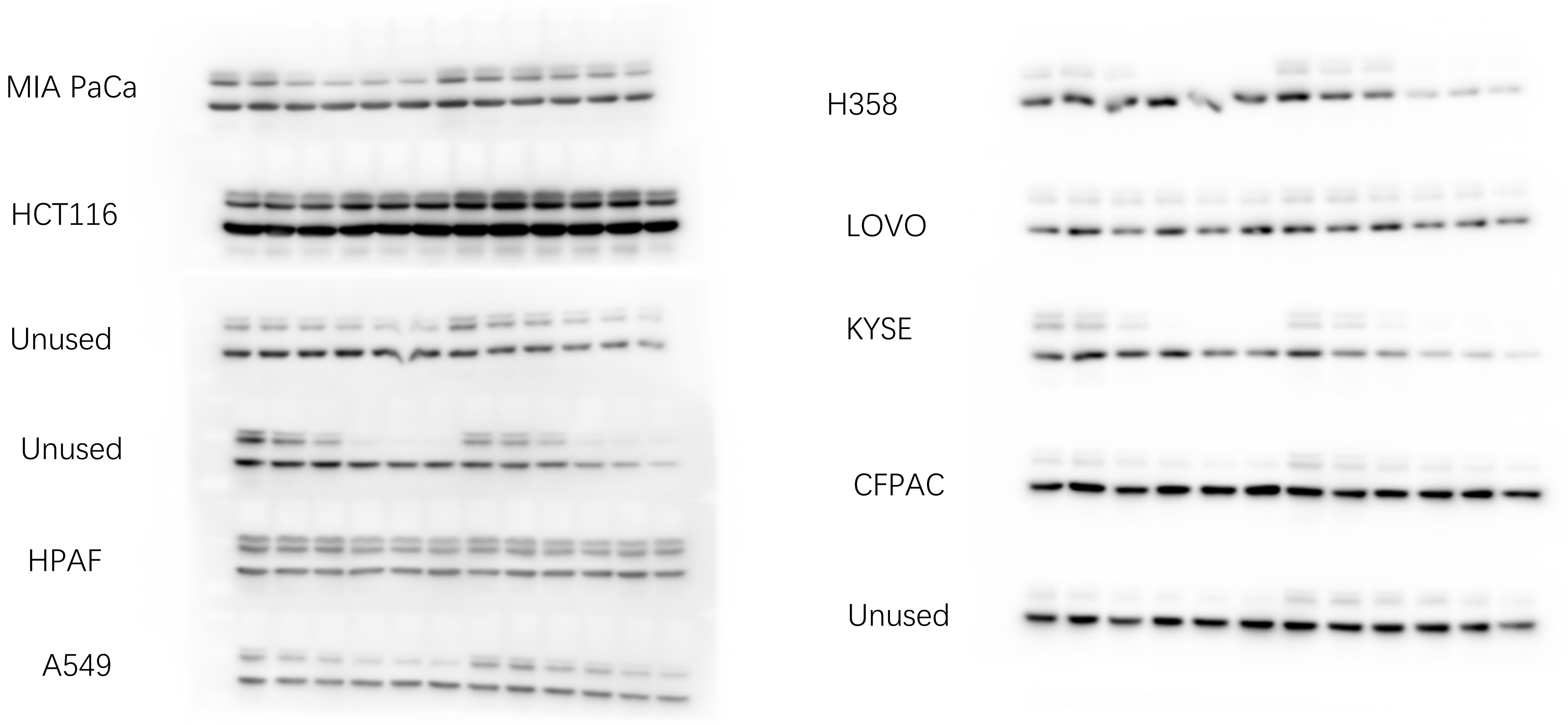

Full unedited gel for Supplemental Figure S3B
LC3

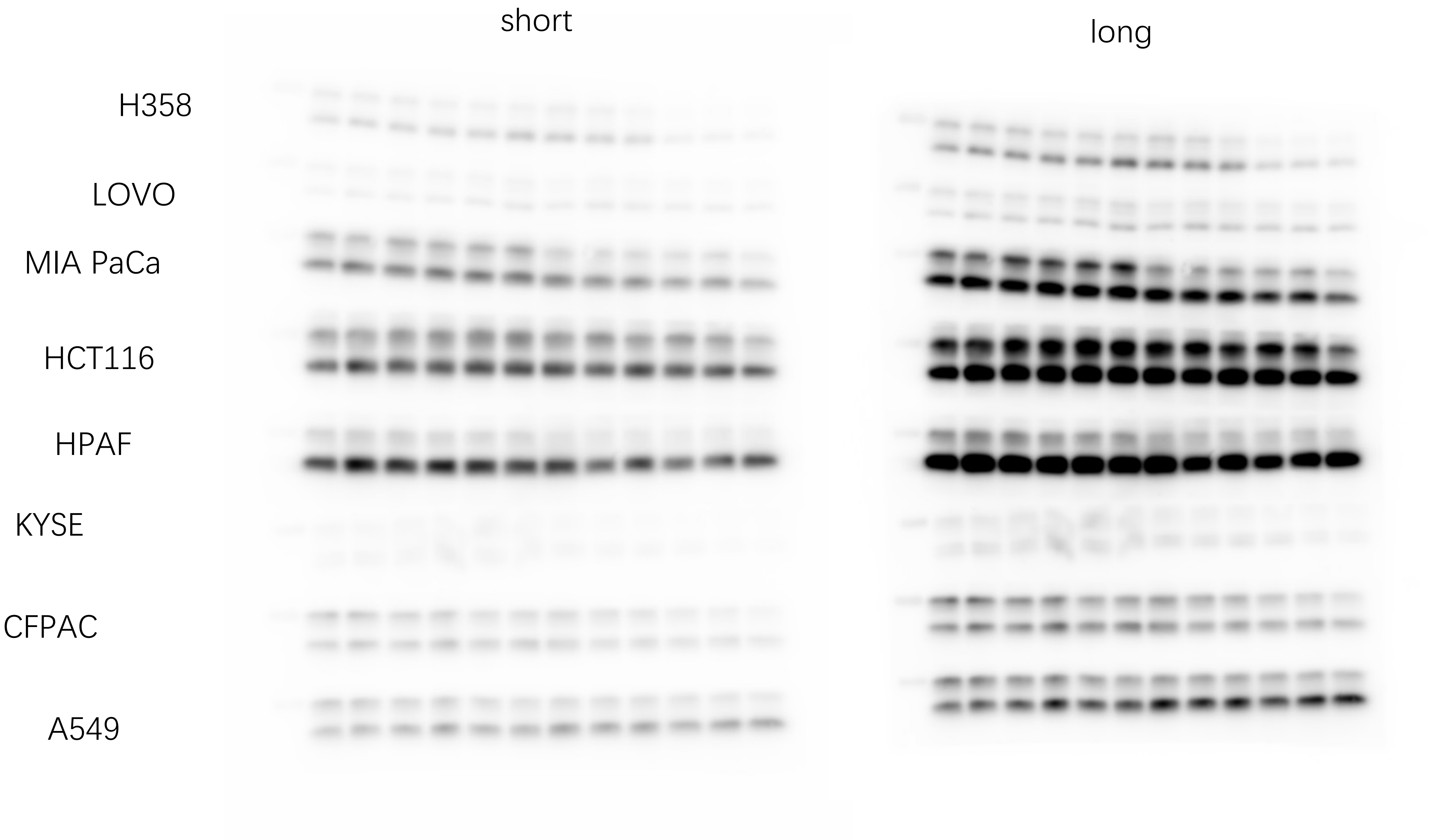

Full unedited gel for Supplemental Figure S3C

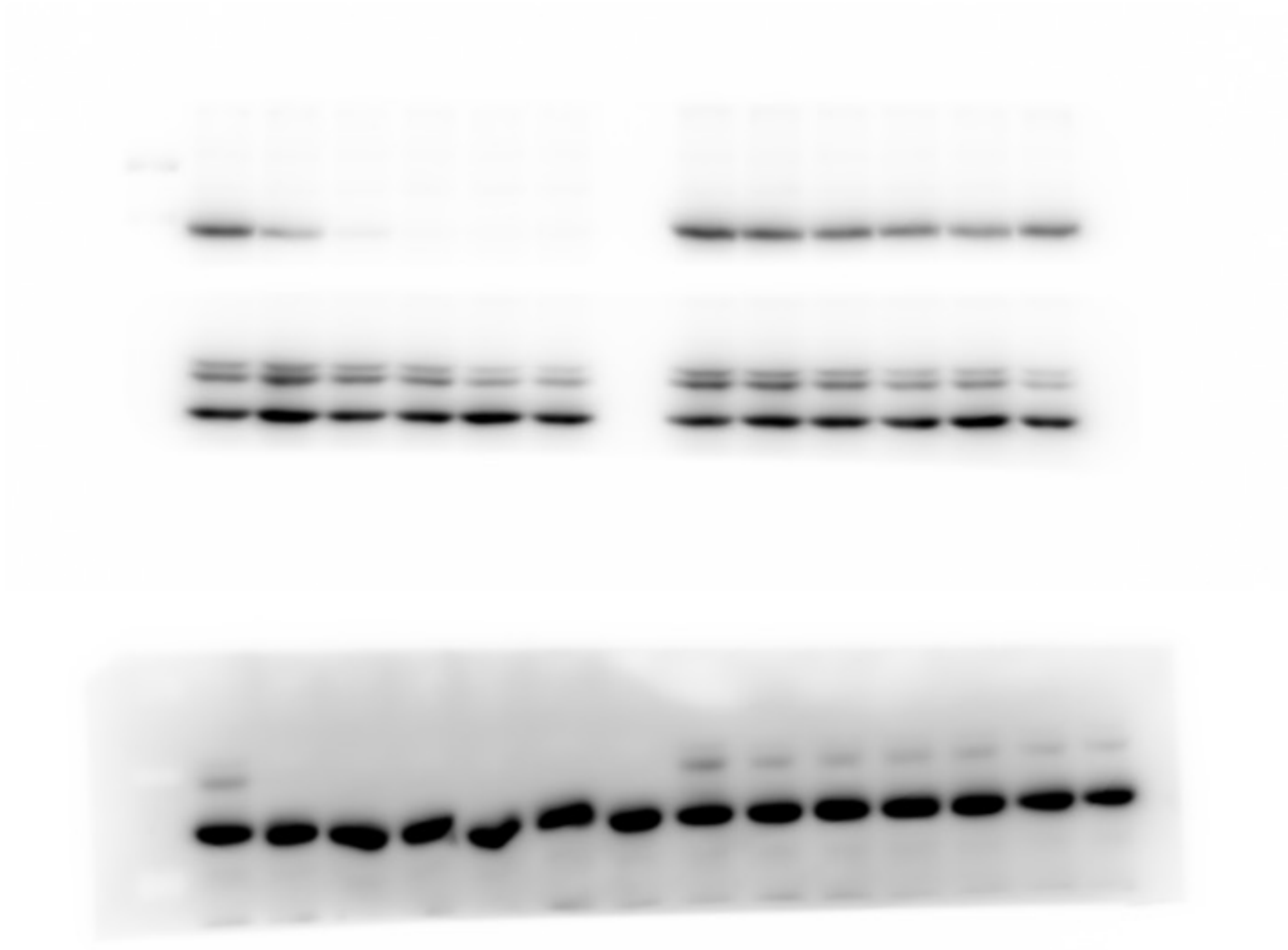

Full unedited gel for Supplemental Figure S5B

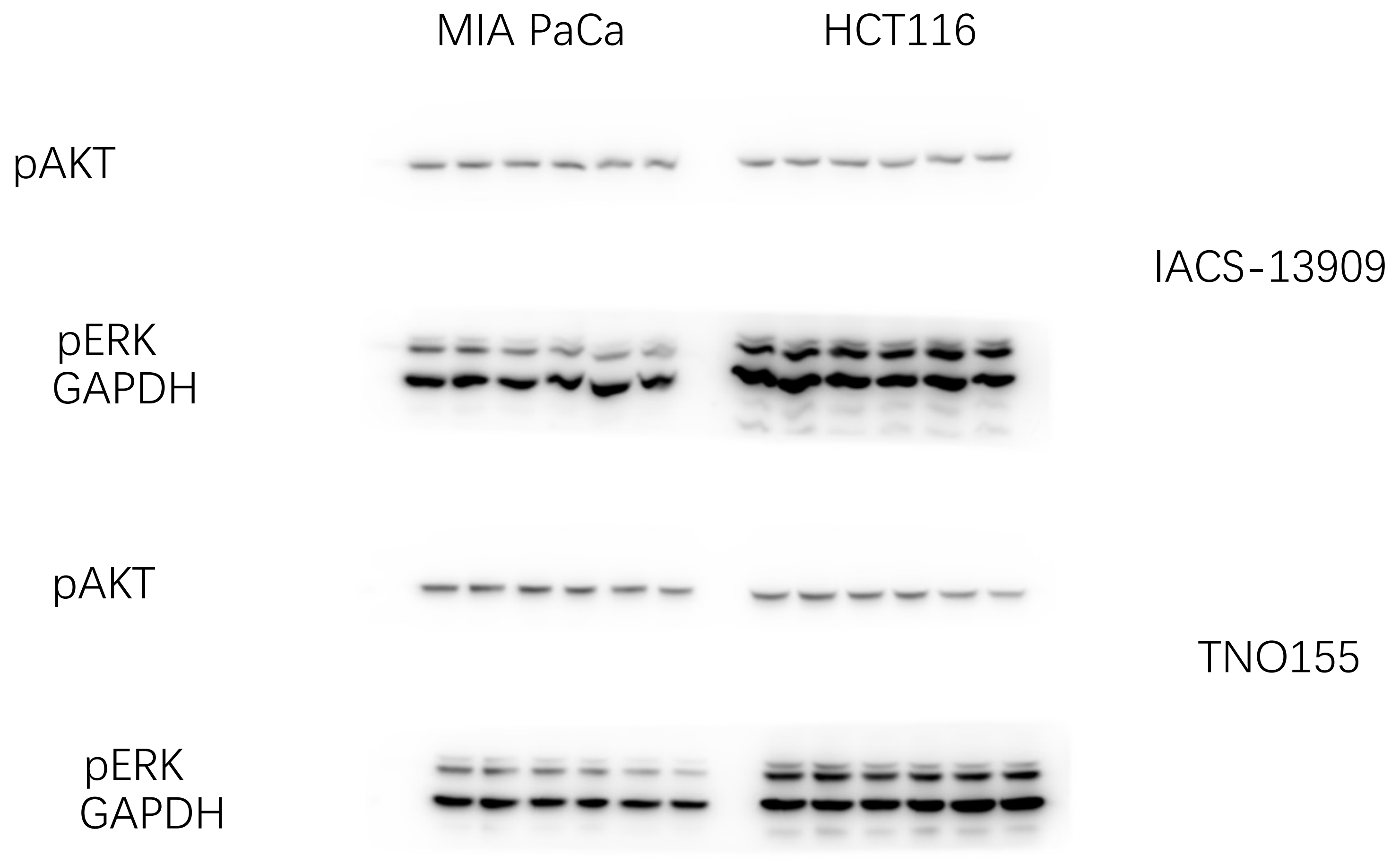

Supplement: Unedited blot and gel images [file jci-134-177142-s246.pdf]
